# Identification of Chemical Constituents from Leaves and Stems of *Alpinia oxyphylla*: Potential Antioxidant and Tyrosinase Inhibitory Properties

**DOI:** 10.3390/antiox13121538

**Published:** 2024-12-16

**Authors:** Huiqin Chen, Xin Su, Pan Xiang, Yanmei Wei, Hao Wang, Juntao Li, Shoubai Liu, Wenli Mei, Haofu Dai

**Affiliations:** 1 Hainan Key Laboratory of Research and Development of Natural Product from Li Folk Medicine, Institute of Tropical Bioscience and Biotechnology, Chinese Academy of Tropical Agricultural Sciences, Haikou 571101, China; chenhuiqin@itbb.org.cn (H.C.); suxinnpc@126.com (X.S.); weiyanmei@itbb.org.cn (Y.W.); wanghao@itbb.org.cn (H.W.); lijuntao@itbb.org.cn (J.L.); 2 Hainan Institute for Tropical Agricultural Resources, Haikou 571101, China; 3 Zhongshan Institute for Drug Discovery, Shanghai Institute of Materia Medica, Chinese Academy of Sciences, Zhongshan 528400, China; xiangpan284@zidd.ac.cn; 4 Key Laboratory of Genetics and Germplasm Enhancement in Tropical Specific Forest Trees and Ornamental Plants, Ministry of Education, Hainan Key Laboratory for Biology of Tropical Specific Ornamental Plants Germplasm, School of Tropical Agriculture and Forestry, Hainan University, Haikou 570228, China; liushoubai@hainanu.edu.cn

**Keywords:** *Alpinia oxyphylla*, tyrosinase inhibitor, antioxidant, melanin production inhibitor

## Abstract

*Alpinia oxyphylla* Miq. is an important undergrowth species in southern China. The fruits of *A. oxyphylla* are recognized as one of “the four famous south medicines” and are also used in the production of preserved fruit. However, as non-medicinal parts, their stems and leaves are unutilized. In order to promote resource recycling, the chemical components of such stems and leaves were investigated, and we evaluated their melanin inhibitory potential through DPPH and ABTS radical scavenging, tyrosinase inhibition, and melanin production inhibition in B16 cells. Five new compounds, aloxy A (**1**), kaempferol 3-*O*-*α*-L-rhamnosyl-(1 → 2)-(3″,4″-diacetyl-*β*-D-glucuronate methyl ester) (**2**), quercetin 3-*O*-*α*-L-rhamnosyl-(1 → 2)-(3″,4″-diacetyl-*β*-D-glucuronate methyl ester) (**3**), kaempferol 3-*O*-*α*-L-rhamnosyl-(1 → 3)-(4″-acetyl-*β*-D-glucuronate methyl ester) (**4**), and kaempferol 3-*O*-*α*-L-rhamnosyl-(1 → 2)-(3″-acetyl-*β*-D-glucuronate methyl ester) (**5**), and seventeen known ones (**6**–**22**) were isolated and identified from the stems and leaves of *A. oxyphylla*. Among these compounds, 19 compounds presented tyrosinase inhibitory activities, among which aloxy A (**1**), hexahydrocurcumin (**7**), gingerenone A (**8**) and 4,4′-dimethoxy-3′-hydroxy-7,9′:7′,9-diepoxylignan-3-*O*-*β*-D-glucopyranoside (**22**) showed strong inhibitory activity, with IC_50_ values between 6.26 ± 0.42 and 22.04 ± 1.09 μM, lower than the positive control (Kojic acid, IC_50_ = 37.22 ± 1.64 μM). A total of 15 compounds exhibited varying degrees of DPPH and ABTS radical scavenging activities. In addition, **1**, **2**, and **7** showed melanin production inhibition activity in B16 cells, and the effects presented as concentration-dependent. The above results indicate that the stems and leaves of *A. oxyphylla* are rich with phenolic compounds, and display tyrosinase inhibition and antioxidant activities, which could lead to potential applications related to melanin production inhibition such as in the development of cosmetics.

## 1. Introduction

Melanin, a pigmented polymer, not only determines the colour of human skin, hair, and eyeballs, but also serves as a protective barrier against ultraviolet radiation [1]. However, overproduction of melanin can increase the risk of skin disorders such as freckles, chloasma, solar lentigo, and age spots [2]. The production of melanin begins with the oxidation of tyrosine to dopaquinone, catalyzed by tyrosinase. Dopaquinone subsequently undergoes auto-oxidation to form 3,4-dihydroxyphenylalanine (dopa) and dopachrome. A series of enzymatic reactions then convert these intermediates into 5,6-dihydroxyindole (DHI) and 5,6-dihydroxyindole carboxylic acid (DHICA), which finally polymerize to form eumelanins, the type of melanin predominantly responsible for brown and black pigmentation [3,4,5]. As a rate-limiting enzyme, tyrosinase is a critical target for inhibiting melanin production. Many skin-whitening products contain tyrosinase inhibitors, such as vitamin C, kojic acid, and arbutin [1]. However, due to their instability and potential adverse health effects, there is a growing interest in plant-original compounds with tyrosinase inhibitory activity. Notable examples include stilbenes (e.g., oxyresveratrol) [6], phenolic acids (e.g., ferulic acid) [7], and flavonoids (e.g., cudraflavone A) [8]. Additionally, exposure to UV radiation and harmful chemicals can stimulate melanocytes to produce oxygen free radicals, such as superoxide anion radicals, superoxide, and singlet oxygen. These free radicals can induce melanocyte proliferation and release of α-melanocyte-stimulating hormone, further contributing to excessive melanin production [2,8]. Therefore, tyrosinase inhibitors and antioxidants are crucial in the cosmetic industry and in the treatment of pigment-related disorders.

*Alpinia oxyphylla* Miq., a perennial Zingiberaceae plant, is widely distributed in southern China. It is especially widely cultivated in Hainan province, where it serves as an economically valuable understory plant. The fresh unripe fruits of *A. oxyphylla* are utilized in the commercial production of preserved fruit products such as “jiu zhi yi zhi” and “mi jian yi zhi”; they are also consumed directly when pickled with soy sauce in traditional practises. According to Chinese Pharmacopeia (2020 edition), the ripe fruits are employed in traditional Chinese medicine for warming the spleen and kidneys, as well as for alleviating diarrhea and excessive saliva. Previous phytochemical studies have reported that the main constituents of *A. oxyphylla* include sesquiterpenes [9,10,11], diarylheptanoids [12], flavones [12], and nucleosides [13]. Moreover, pharmacological studies have demonstrated that *A. oxyphylla* exhibits multiple pharmacological activities, including neuroprotective [11], anti-inflammatory, anti-nociceptive [14], and anti-diuretic effects [15]. Despite these benefits, utilization is currently limited to the fruits; abundant quantities of leaves and stems are discarded, which not only wastes resources, but also causes environmental pollution. A study of the roots, fruits, and leaves of *A. oxyphylla* carried out using UPLC-MS/MS revealed differences in composition among the different plant parts. Fruits were found to be abundant in flavonoids, while the roots contained high contents of flavonoids and phenolic acids, and the leaves were rich in flavonoids, phenolic acids, and tannins. Network pharmacology analysis has further indicated that these different tissues possess varying medicinal values [16]. In a previous study, we demonstrated that the ethanol extract obtained from the stems and leaves of *A. oxyphylla* exhibited a growth-promoting effect, altered muscle composition, and provided antioxidative stress benefits in juvenile *Litopenaeus vannamei* [17]. In the present work, we aimed to further explore the potential utilization of the parts of *A. oxyphylla* which are often discarded; specifically, the leaves and stems. To this end, their chemical components were separated using various chromatographic techniques. The identified compounds were then screened for their tyrosinase inhibitory activity, and their antioxidant activities. DPPH and ABTS radical scavenging tests were carried out, and the melanin inhibitory effect on B16 cells was also considered. The results of this study provide a comprehensive evaluation of the potential applications of *A. oxyphylla* leaves and stems for use in cosmetic formulations and in the development of medicines for treatment of pigmentation disorders.

## 2. Materials and Methods

### 2.1. General Experimental Procedures

Optical rotation was measured on an Anton Paar MCP 5100 polarimeter (Anton Paar, Graz, Austria). HRESIMS was recorded on a Bruker Compact mass spectrometer (Bruker, Bremen, Germany). NMR spectra were measured on a Bruker AVIII spectrometer (Bruker, Bremen, Germany) at 500 MHz (^1^H NMR) and 125 MHz (^13^C NMR). Analytic high-performance liquid chromatography (HPLC) was conducted on an Agilent Technologies 1260 Infinity (Agilent, Palo Alto, CA, USA) equipped with a YMC-packed C_18_ column (250 mm × 4 mm, 5 μm, flow rate = 1 mL/min). Semi-preparative HPLC was prepared on a YMC-packed C_18_ column (250 mm × 10 mm, 5 μm, flow rate = 4 mL/min). Silica gel (60–80 mesh, Qingdao Haiyang Chemical Co., Ltd., Qingdao, China), ODS gel (20–45 μm, Fuji Silysia Chemical Co., Ltd., Nagoya, Japan, and Sephadex LH-20 (Merck, Darmstadt, Germany) were used for column chromatography.

### 2.2. Plant Materials

Fresh leaves and stems of *A. oxyphylla* were provided by Prof. Xiao-Xia Yan and Mao-Yuan Wang, Tropical Crops Genetic Resource Institute, Chinese Academy of Tropical Agricultural Sciences (Hainan Province in China), in June 2020. The plant material was identified as *Alpinia oxyphylla* Miq. by Dr. Shoubai Liu from Hainan University. A voucher specimen (No. AO20200620) was deposited in the Institute of Tropical Bioscience and Biotechnology, Chinese Academy of Tropical Agricultural Sciences.

### 2.3. Extraction and Isolation

Fresh leaves and stems (95.5 Kg) were reflux extracted with 95% ethanol (keep faint boiling for 1 h), and the filtrate was concentrated until no ethanol smell remained. The residue was removed, and the solution was liquid–liquid separated with ethyl acetate (EtOAc) and *n*-butanol (*n*-BuOH) successively to obtain EtOAc fraction (150.1 g) and *n*-BuOH fraction (348.4 g). Subsequently, EtOAc fraction was submitted to a silica gel vacuum liquid column (VLC) eluted with chloroform (CHCl_3_)–methanol (MeOH) (1:0 to 1:1, *v*/*v*) to afford 20 fractions (Fr.1–Fr.20).

Fr.2 (2.03 g) was subjected to Sephadex LH-20 column chromatography eluted with CHCl_3_: MeOH (1:1, *v*/*v*) to obtain 5 subfractions. Fr.2.1 (662.1 mg) was fractionated by silica gel CC with petroleum ether (PE): EtOAc (500:1 to 20:1, *v*/*v*) to obtain 30 fractions. Fr.2.1.22 (65.7 mg) was rechromatographed over silica gel CC with PE: EtOAc (40:1, *v*/*v*) to yield compound **22** (4.2 mg). Fr.2.2 (124.0 mg) was separated by silica gel CC eluted with PE: EtOAc (130:1, *v*/*v*) to give compound **6** (6.6 mg). Fr.2.3 (322.8 mg) was firstly separated by silica gel CC using PE: EtOAc (150:1 to 140:1, *v*/*v*) as eluent to obtain 25 fractions. Fr.2.3.20 (11.5 mg) was washed with methanol, and the residue was compound **13** (7.5 mg).

Fr.3 (2.20 g), Fr.16 (5.64 g) and Fr.16 (9.77 g) were divided into 3, 8, and 9 fractions, respectively, by Sephadex LH-20 eluting by CHCl_3_: MeOH (1:1, *v*/*v*). And then, Fr.3.1 (141.8 mg) and Fr.4.8 (107.4 mg) were submitted to silica gel CC eluting with PE: EtOAc (10:1, *v*/*v*), respectively. Compound **12** (11.6 mg) was isolated from Fr.3.1, while compounds **14** (3.1 mg), **7** (3.5 mg), and **8** (4.0 mg) were obtained from Fr.4.8. Fr. 16.8 (455.9 mg) was further separated by silica gel CC with gradient CHCl_3_: MeOH (12:1 to 10:1, *v*/*v*) as eluent to obtain 12 fractions. Fr.12.8.4 (19.2 mg) was purified by preparative TLC to yield **19** (13.0 mg).

Fr.15 (4.11 g) was separated into 20 fractions (Fr.15.1–20) by silica gel CC. Subsequently, Fr.15.1 (90.9 mg), Fr.15.2 (690.0 mg), and Fr.15.3 (830.0 mg) were chromatographed over Sephadex LH-20 CC to give 16, 13, and 7 fractions, respectively. Fr.15.1.5 (13.4 mg) was separated by preparative TLC to afford **17** (6.5 mg) and **18** (8.7 mg). Fr.15.2.10 was isolated by semi-preparative HPLC (MeOH: H_2_O = 43:57, *v*/*v*; flow rate = 4 mL/min) to yield **15** (3.5 mg, *t*_R_ = 27.0 min). Fr.15.3.2 (243.2 mg) was applied to silica gel CC eluted with CHCl_3_: MeOH (50:1 to 40:1, *v*/*v*) to give 12 fractions. Fr.15.3.2.5 (21.5 mg) was recrystallized to produce **16** (3.8 mg). Fr.15.3.2.9 (21.2 mg) was separated by semi-preparative HPLC to yield **1** (8.5 mg, *t*_R_ = 24.2 min).

**Figure 1:**
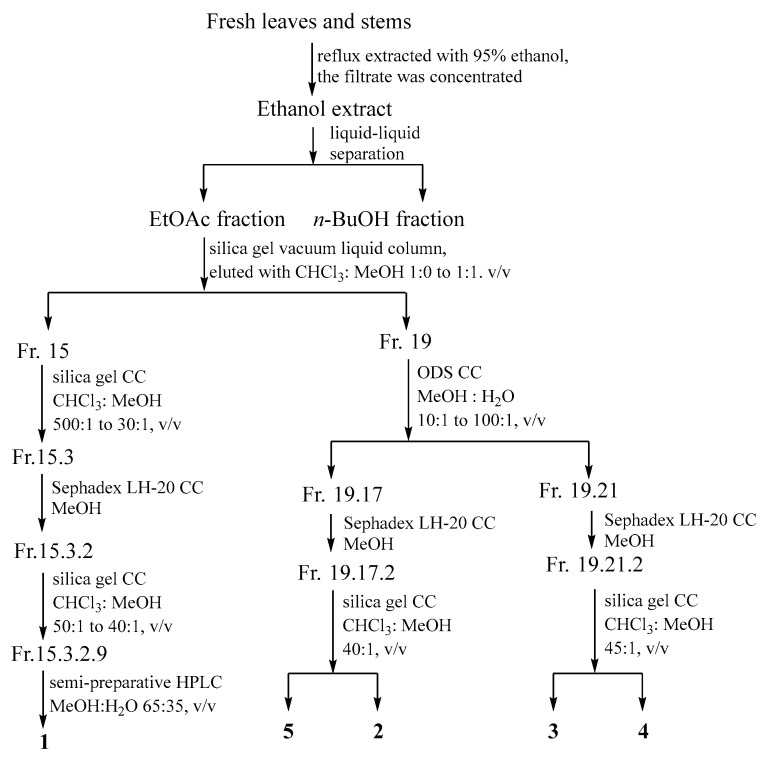
Isolation process of 5 new compounds.

Fr.19 (8.84 g) was fractionated by ODS CC to obtain 22 fractions, together with compounds **20** (4.8 mg) and **21** (7.3 mg). Fr.19.17 (993.3 mg), Fr.19.18 (498.5 mg), and Fr.19.21 (590.8 mg) were submitted to Sephadex LH-20 CC to obtain 10, 5, and 2 fractions, respectively. Fr.19.17.2 (143.0 mg) was separated by silica gel CC to obtain compounds **5** (5.9 mg) and **2** (2.4 mg). Fr.19.17.8 (23.2 mg) was subjected to silica gel to obtain **10** (4.7 mg). Fr.19.18.3 (124.1 mg) and Fr.19.18.5 (80.5 mg) were separated by silica gel CC to yield **11** (1.9 mg) and **9** (15.4 mg), respectively. Fr.19.21.2 (137.5 mg) was applied to silica gel CC to yield **3** (5.2 mg) and **4** (2.4 mg) (Figure 1).

Aloxy A (**1**): Colourless oil; [*α*]^D^_25_ −21.0 (*c* 0.10, CH_3_OH); HRESIMS, *m*/*z* 861.3341 [M + Na]^+^ (calc. for C_44_H_54_O_16_Na, 861.3304); ^1^H and ^13^C data shown in Table S1.

Kaempferol 3-*O*-*α*-L-rhamnosyl-(1 → 2)-(3″,4″-diacetyl-*β*-D-glucuronate methyl ester) (**2**): Yellow crystals; [*α*]^D^_25_ −54.0 (*c* 0.10, CH_3_OH); HRESIMS, *m*/*z* 729.1638 [M + Na]^+^ (calcd. for C_32_H_34_O_18_Na, 729.1637); ^1^H and ^13^C data shown in Tables S2 and S3.

Quercetin 3-*O*-α-L-rhamnosyl-(1 → 2)-(3″,4″-diacetyl-*β*-D-glucuronate methyl ester) (**3**): Yellow crystals; [*α*]^D^_25_ −38.0 (*c* 0.10, CH_3_OH); HRESIMS, *m*/*z* 745.1599 [M + Na]^+^ (calcd. for C_32_H_34_O_19_Na, 745.1586); ^1^H and ^13^C data shown in Tables S2 and S3.

Kaempferol 3-*O*-*α*-L-rhamnosyl-(1 → 3)-(4″-acetyl-*β*-D-glucuronate methyl ester) (**4**): Amorphous powder; [*α*]^D^_25_ −43.0 (*c* 0.10, CH_3_OH); HRESIMS, *m*/*z* 687.1538 [M + Na]^+^ (calcd. for C_30_H_32_O_17_Na, 687.1532); ^1^H and ^13^C data shown in Tables S2 and S3.

Kaempferol 3-*O*-*α*-L-rhamnosyl-(1 → 2)-(3″-acetyl-*β*-D-glucuronate methyl ester) (**5**): Amorphous powder; [*α*]^D^_25_ −52.0 (*c* 0.10, CH_3_OH); HRESIMS, *m*/*z* 687.1554 [M + Na]^+^ (calcd. for C_30_H_32_O_17_Na, 687.1532); ^1^H and ^13^C data shown in Tables S2 and S3.

### 2.4. X-Ray Crystallographic Data of ***2***

Crystal Data for **2**: C_32_H_34_O_18_ (M = 706.17 g/mol): orthorhombic, space group P2_1_2_1_2_1_ (no. 19), *a* = 14.06350 (10) Å, *b* = 21.49840 (10) Å, *c* = 24.1589 (2) Å, *V* = 7304.27 (9) Å3, *Z* = 8, *T* = 170.00 (10) K, μ (Cu Kα) = 1.034 mm^−1^, *Dcalc* = 1.400 g/cm^3^, 91,103 reflections measured (5.502° ≤ 2Θ ≤ 147.302°), 14,582 unique (R_int_ = 0.0354, R_sigma_ = 0.0196), which were used in all calculations. The final R_1_ was 0.0277 (I > 2σ(I)) and *w*R_2_ was 0.0711. Crystallographic data (excluding structure factors) for structure **2** in this paper have been deposited in the Cambridge Crystallographic Data Center under supplementary publication number CCDC2312077.

### 2.5. DPPH Radical Scavenging Activity Assay

The DPPH radical scavenging capacity was measured according to the following reference at room temperature (25 °C) [18]. Briefly, 320 μL sample in DMSO with different concentrations (400, 200, 100, 50, 25, 12.5 μM) was reacted with 320 μL DPPH reagent (0.1 mM) in 1.5 mL centrifuge tube away from light for 30 min. Next, 200 μL of the mixture was transferred into a 96-well plate, which was used for measuring the absorbance at 517 nm (OD_sample_). A control (OD_control_) and blank (OD_blank_) were, respectively, established from the mixture solutions of the sample in DMSO and ethanol and the mixture solution of DMSO and DPPH reagent. Vitamin C was the positive control, with the same concentration as the compounds. The DPPH radical scavenging capacity was calculated according to the following:

DPPH radical scavenging capacity (%) = [1 − (OD_sample_ − OD_control_)/OD_blank_] × 100%

### 2.6. ABTS Radical Scavenging Activity Assay

The ABTS radical scavenging activity was examined as previously reported at room temperature (25 °C) [18,19]. Briefly, 160 μL sample in DMSO with different concentrations (800, 400, 200, 100, 50, 25 μM) was reacted with 480 μL ABTS reagent in a 1.5 mL centrifuge tube away from light for 6 min. Next, 200 μL of the mixture was taken out and transferred into a 96-well plate, which was used for measuring the absorbance by a microplate reader at 734 nm (OD_sample_). The control (OD_control_) and blank (OD_blank_) were, respectively, established from the mixture solutions of the sample in DMSO and water and the mixture solution of DMSO and ABTS reagent. Trolox was the positive control, with the same concentration with compounds. The ABTS radical scavenging capacity was calculated as following:

ABTS radical scavenging capacity (%) = [1 − (OD_sample_ − OD_control_)/OD_blank_] × 100%

### 2.7. Theoretical Calculation

The energies of the Highest Occupied Molecular Orbitals (HOMOs) and Lowest Unoccupied Molecular Orbitals (LUMOs) were calculated using the B3LYP/def2-SVP in the Gaussian 16 programme suite. Geometry optimizations without symmetry restriction were carried out with the Gaussian 16 programme [20]. Specifically, geometry optimization corresponding to the unsimplified experimental compounds was firstly performed at the B3LYP [21,22,23]/def2-SVP [24,25] level with Grimme’s GD3BJ [26,27] dispersion corrections. Considering the solvation effects of the solvent used in the experiments (i.e., water), the SMD [28] solvation model was utilized. Vibrational frequency calculations were performed for all stationary points to identify the local minima (no imaginary frequencies) and to derive the thermochemical corrections for the enthalpies and free energies.

### 2.8. Tyrosinase Inhibitory Activity Assay

Tyrosinase inhibitory activity was tested according to the method described by Xie et al. at room temperature (25 °C) (2022) [29] with slight modification. In brief, 585 μL tyrosinase solution (50 U/mL) was placed in a 1.5 mL centrifuge tube, and then mixed with 90 μL sample in MeOH/H_2_O 1:1 with different concentrations (2 Mm, 1 mM, 0.5 mM, 0.25 mM, 0.125 mM, 0.0625 mM). Next, it was transferred into 150 μL mixture solution into a 96-well plate, to which was added 50 μL tyrosine solution (2 mM) after 15 min at 37 °C. Kojic acid instead of the sample was set as the positive control, with the same concentration with the compounds. PBS solution instead of tyrosine solution was set as the blank, and the solvent MeOH/H_2_O 1:1 instead of the sample was set as the negative control. After another 30 min, the absorbance (OD) was measured. The tyrosinase inhibitory activity was calculated as follows:

Tyrosinase inhibitory rate (%) = (OD_negative control_ − OD_sample_)/(OD_negative control_ − OD_blank_) × 100%

### 2.9. Cell Culture

B16 melanoma cells (PNS-MC-36) were purchased from Procell Life Science & Technology Co., Ltd. (Wuhan, China). The cells were cultivated in RPMI-1640 medium supplemented with 10% fetal bovine serum in the following conditions: 37 °C, 5% CO_2_, and 90% humidity.

### 2.10. MTT Assay for Cell Viability

A modified MTT method was applied to screen cell viability [30]. B16 cells were cultured in 96-well plates and mixed with different concentrations of the sample after 24 h. After having been incubated for another 72 h, MTT solution was added to form formazan, which was followed by dissolved in 100 μL DMSO, and then the absorbance (OD) was measured at 490 nm. Cell viability was calculated as percent changes relative to control.

### 2.11. Measurement of Melanin Produced by B16 Cells

B16 cells were seeded into 96-well plates with 4 × 10^5^ cells/well, and incubated at 37 °C, 5% CO_2_, and 90% humidity for 24 h. Having removed the supernatant, 100 μL samples with different concentrations were added. Medium without the sample was added as the blank. After incubation for another 72 h, the cells were dissolved in 100 μL of 2 N NaOH, and then incubated at 80 °C. The absorbance (OD) was measured at 450 nm.

## 3. Results and Discussion

### 3.1. Evaluation of Activity of Extracts from Leaves and Stems of A. oxyphylla

Ethanol extract was obtained from the leaves and stems of *A. oxyphylla*, and a bioactivities assay was carried out. The results showed that the ethanol extract exhibited antioxidant activities, with a DPPH free radical scavenging rate of 40.7% (the inhibitory rate of vitamin C was 72.7%), and an ABTS free radical scavenging rate of 90.4% (the inhibitory rate of trolox was 91.6%) at a concentration of 100 μg/mL. Measurements of tyrosinase inhibitory activity showed that the ethanol extract had an inhibitory rate of 74.7% at a concentration of 200 μg/mL (the inhibitory rate of kojic acid was 99.8%).

### 3.2. Isolation of Compounds and Elucidation of Structures

**Figure 2:**
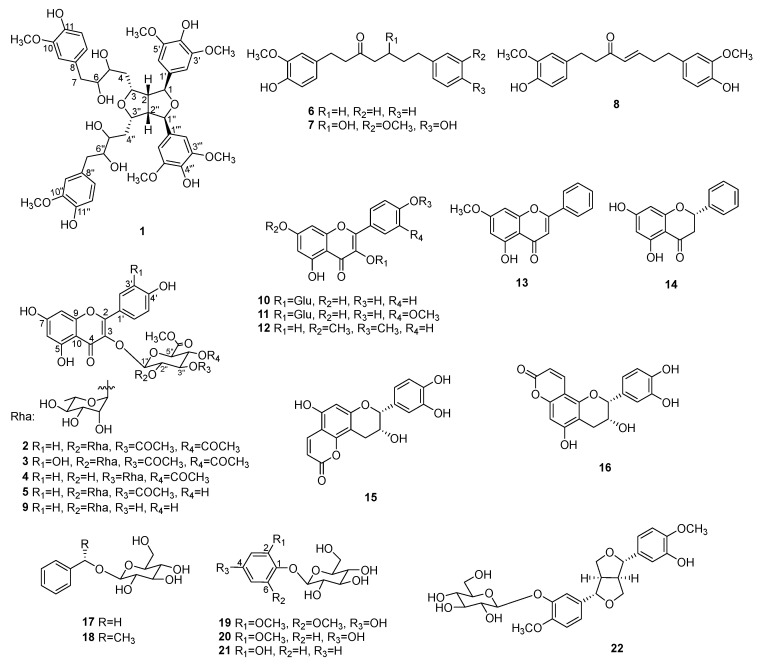
Structures of isolated compounds.

Five new compounds (**1**–**5**) and seventeen knowns (**6**–**22**) (Figure 2) were isolated from EtOAc-soluble ethanol extract of *A. oxyphylla* leaves and stems using various types of column chromatography.

**Figure 3:**
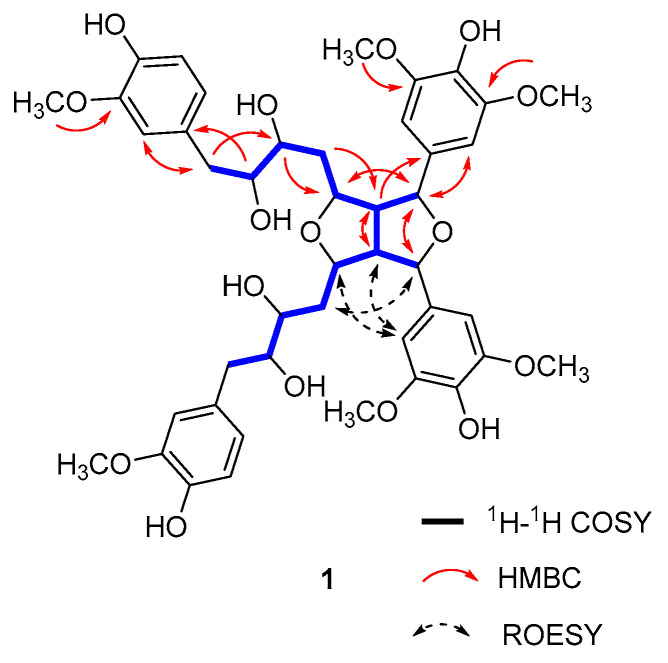
The key ^1^H-^1^H COSY, HMBC, and ROESY correlations of compound **1**.

Compound **1** was isolated as a colourless oil. Its HRESIMS showed a pseudomolecular ion peak at *m*/*z* 861.3341 (calc. for C_44_H_54_O_16_Na, 861.3304), giving the molecular formula of C_44_H_54_O_16_ with 18 degrees of unsaturation. Careful analysis of the spectra revealed that there were 27 protons and 22 carbons presented in the ^1^H and ^13^C NMR, respectively; the numbers were half those detected using HRESIMS. Therefore, a symmetrical structure of diarylheptanoid dimer was deduced. Accordingly, the ^1^H and ^13^C NMR spectra (Table S1) revealed the presence of two ABX coupling aromatic rings [*δ*_C_ 132.1 (C-8, 8″), *δ*_C_ 114.0/*δ*_H_ 6.70 (2H, d, *J* = 1.8 Hz, H-9, 9″), *δ*_C_ 148.7 (C-10, 10″), *δ*_C_ 145.7 (C-11, 11″), *δ*_C_ 116.0/*δ*_H_ 6.65 (2H, d, *J* = 8.0 Hz, H-12, 12″), *δ*_C_ 122.8/*δ*_H_ 6.54 (2H, dd, *J* = 8.0, 1.8 Hz, H-13, 13″)], two meta-unsubstituted aromatic rings [*δ*_C_ 135.0 (C-1′, 1′′′), *δ*_C_ 105.4/*δ*_H_ 6.69 (4H, br s, H-2′, 2′′′, 6′, 6′′′), *δ*_C_ 149.4 (C-3′, 3′′′, 5′, 5′′′), *δ*_C_ 136.3 (C-4′, 4′′′)], four methylenes [*δ*_C_ 36.6/*δ*_H_ 2.05 (2H, dt, *J* = 13.9, 8.0 Hz, H-4a, 4″a), 1.82 (2H, ddd, *J* = 13.9, 8.0, 4.0 Hz, H-4b, 4″b); *δ*_C_ 40.5/*δ*_H_ 2.55 (2H, m, H-7a, 7″a), 2.51 (2H, m, H-7b, 7″b)], ten methines [*δ*_C_ 81.5/*δ*_H_ 5.12 (2H, d, *J* = 6.0 Hz, H-1, 1″), *δ*_C_ 58.6/*δ*_H_ 3.33 (2H, m, overlapped, H-2, 2″), *δ*_C_ 79.9/*δ*_H_ 4.73 (2H, td, *J* = 7.0, 2.9 Hz, H-3, 3″), *δ*_C_ 72.7/*δ*_H_ 3.26 (2H, m, H-5, 5″), *δ*_C_ 76.1/*δ*_H_ 3.47 (2H, m, H-6, 6″)], and six methoxyl groups [*δ*_C_ 56.3/*δ*_H_ 3.76 (6H, s, 10-OCH_3_, 10″-OCH_3_), *δ*_C_ 56.8/*δ*_H_ 3.80 (12H, s, 3′-OCH_3_, 5′-OCH_3_, 3′′′-OCH_3_, 5′′′-OCH_3_)], which were supported by DEPT and HSQC spectra. Moreover, the ^1^H-^1^H COSY correlations (Figure 3) of H-1/H-2/H-3/H_2_-4/H-5/H-6/H_2_-7, together with the key HMBC correlations from H-1 to C-2′ and C-6′, from H-2 to C-1′, from H-6 to C-8, and from H-7 to C-9 and C-13 indicated a diarylheptanoid fragment [31] in the structure. Further HMBC correlations from 10-OCH_3_ to C-10, from 3′-OCH_3_ to C-3′, and 5′-OCH_3_ to C-5′ revealed methoxyl groups located at C-10, C-3′, and C-5′.

Four aromatic rings occupied 16 degrees of unsaturation; therefore, two remaining unsaturations were from two rings, which both consisted of a connection of two diarylheptanoid fragments. Further HMBC correlations from H-1 to C-1′′′, and from H-2 to C-2′′′, confirmed that bitetrahydrofuran was formed between two diarylheptanoids. Moreover, the observed ROESY correlations of H-1/H-4 and H-2/H-2′/H-3 revealed that H-2, H-2′′′, H-3, and H-3′′′ were cofacial, while H-1 and H-1′′′ were located at the opposite face. Unfortunately, because we failed to obtain a crystal, and the configurations of 4 (4′)-OH and 6 (6)-OH could not be determined. Based on the above, the structure of **1** was established, and the trivial name of aloxy A was given to it.

**Figure 4:**
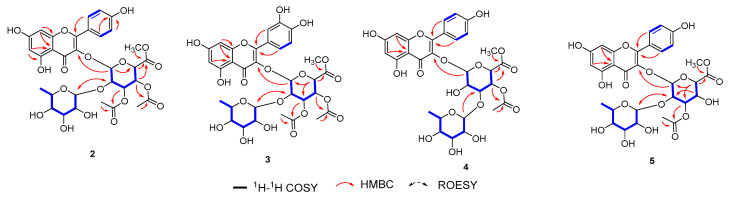
The key ^1^H-^1^H COSY, HMBC, and ROESY correlations of compounds **2**–**5**.

Compound **2** was obtained as a yellow crystal, and the molecular formula was deduced as C_32_H_34_O_18_ by ^13^C NMR and HRSEIMS at the quasimolecular ion peak [M + Na]^+^ at *m*/*z* 729.1638 (calcd. 729.1637). After detailed analysis of ^1^H and ^13^C NMR, it was proposed that **1** was a flavonoid glycoside. The aglycone of **1** was identified as kaempferol [32] according to a *meta*-unsubstituted aromatic ring [*δ*_C_ 163.1 (C-5), *δ*_H_ 6.17 (1H, d, *J* = 2.1 Hz, H-6)/*δ*_C_ 99.8, *δ*_C_ 165.8 (C-7), *δ*_H_ 6.37 (1H, d, *J* = 2.1 Hz, H-8)/*δ*_C_ 94.7, *δ*_C_ 158.4 (C-9), *δ*_C_ 105.9 (C-10)], a para-substituted aromatic ring [*δ*_C_ 122.8 (C-1′), *δ*_H_ 8.02 (1H, d, *J* = 8.8 Hz, H-2′, 6′)/*δ*_C_ 132.2, *δ*_H_ 6.92 (1H, d, *J* = 8.8 Hz, H-3′, 5′)/*δ*_C_ 116.2, *δ*_C_ 161.5 (C-4′)], and an α, β-unsaturated ketone [*δ*_C_ 159.1 (C-2), *δ*_C_ 134.0 (C-3), *δ*_C_ 178.7 (C-4)] (Tables S2 and S3). The terminal group at *δ*_H_ 5.90 (1H, d, *J* = 7.5 Hz, H-1″)/*δ*_C_ 100.1, and ^1^H-^1^H COSY correlations (Figure 4) of H-1″/H-2″ (*δ*_H_ 3.90)/H-3″ (*δ*_H_ 5.39)/H-4″ (*δ*_H_ 5.08)/H-5″ (*δ*_H_ 4.23), as well as the HMBC correlations (Figure 4) from H-1″ to C-5″ (*δ*_C_ 73.3) and from H-4″ to C-6″ (*δ*_C_ 169.0) suggested glucoside moiety with methyl esterification of C-6″; this was further confirmed by HMBC correlation of 6″-OCH_3_ (*δ*_H_ 3.58)/C-6″. In addition, acetyl groups were located at 3″-OH and 4″-OH according to the HMBC correlations from H-3″/3″-COCH_3_ (*δ*_H_ 2.11) to 3″-COCH_3_ (*δ*_C_ 171.5), and H-4″/4″-COCH_3_ (*δ*_H_ 1.98) to 4″-COCH_3_ (*δ*_C_ 171.4), respectively. The remaining signals were assigned to rhamnosyl moiety by analyzing 1D and 2D NMR. The connection of C3-O-C1″ and C2″-O-C1′′′ was inferred from the HMBC correlations of H-1″/C-3 and H-1′′′/C-2″.

**Figure 5:**
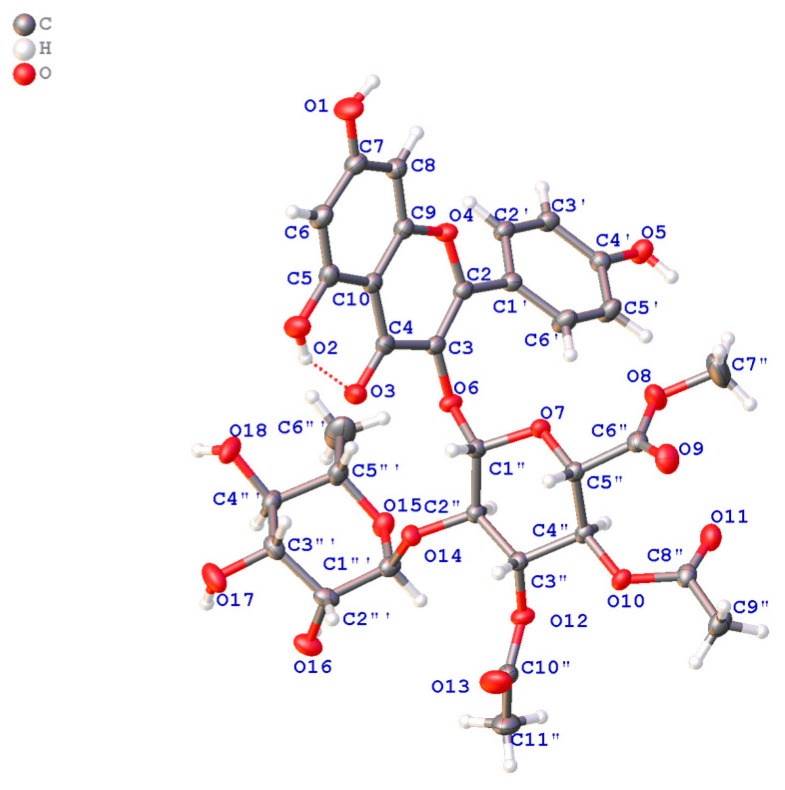
Single-crystal X-ray diffractometry of **2**.

The relative configurations of the glucoside and rhamnosyl moieties were determined using *J* coupling constants and NOESY correlations. The large coupling constants of ^3^*J*_H1″–H2″_ = 7.5 Hz, ^3^*J*_H2″–H3″_ = 8.3 Hz, ^3^*J*_H3″–H4″_ = 9.3 Hz, and ^3^*J*_H4″–H5″_ = 9.7 Hz indicated that H-1″~H-5″ were in axial positions; this was further confirmed by the NOESY correlations of H-3″/H-1″/H-5″ and H-2″/H-4″. A review of the coupling constants of ^3^*J*_H1′′′–H2′′′_ = 5.6 Hz, ^3^*J*_H2′′′–H3′′′_ = 2.1 Hz, ^3^*J*H_3′′′–H4′′′_ = 9.5 Hz, and ^3^*J*_H4′′′–H5′′′_ = 6.1 Hz indicated that all hydrogens except H-2_′′′_ were axial [33]. Fortunately, the crystal of **2** was obtained (Figure 5), so the absolute configuration of **2** could be assigned, as shown in Figure 4. The structure of **2** was thus determined to be kaempferol 3-*O*-*α*-L-rhamnosyl-(1 → 2)-(3″,4″-diacetyl-*β*-D-glucuronate methyl ester).

Compound **3** was acquired as a yellow crystal. The molecular formula was determined to be C_32_H_34_O_19_ on the basis of the pseudomolecular ion peak at *m*/*z* 745.1599, as observed in the HRESIMS spectrum. A detailed comparison of the 1D (Tables S2 and S3) and 2D NMR (Figure 4) spectra of compounds **3** and **2** revealed that the only difference between them involved substitutes on the aromatic ring at the aglycone part, so that there was an ABX coupling system in **3,** instead of the AA′BB′ coupling system in **2**. The typical ^1^H NMR signals of *δ*_H_ 7.60 (1H, d, *J* = 2.2 Hz, H-2′), *δ*_H_ 6.90 (1H, d, *J* = 8.4 Hz, H-5′), and *δ*_H_ 7.58 (1H, dd, *J* = 8.4, 2.2 Hz, H-6′), as well as the downfield chemical shifts of C-3′ (*δ*_C_ 146.0) and C-4′ (*δ*_C_ 149.8), together with the HMBC correlations from H-2′ and H-6′ to C-2 (*δ*_C_ 159.0), revealed one more hydroxyl group located at C-3′, which was consistent with a 16 amu increase in the molecular weight, compared with **2**. Therefore, the structure of 3 was identified as quercetin 3-*O*-*α*-L-rhamnosyl-(1 → 2)-(3″,4″-diacetyl-*β*-D-glucuronate methyl ester).

Compound **4** was obtained as an amorphous powder. The molecular formula of C_30_H_32_O_17_ was obtained from the HRESIMS spectrum at the quasimolecular ion peak [M + Na]^+^ at *m*/*z* 687.1538 (calcd. 687.1532); thus, one acetyl group was missing, compared with **2**. Further comparison of the 1D (Tables S2 and S3) and 2D NMR (Figure 4) spectra of **4** and **2** revealed that the acetyl group at C-3 in **4** disappeared, while the rhamnosyl moiety at C-2″ in **2** was moved to C-3″ in **4**, a result confirmed by HMBC correlation from the terminal proton of rhamnosyl moiety H-1′′′ [*δ*_H_ 5.23 (1H, d, *J* = 1.7 Hz)] to C-3″ (*δ*_C_ 75.7). Hence, compound **4** was identified as kaempferol 3-*O*-*α*-L-rhamnosyl-(1 → 3)-(4″-acetyl-*β*-D-glucuronate methyl ester).

Compound **5** was isolated as amorphous powder. The quasimolecular ion peak (*m*/*z* 687.1554) presented in HRESIMS gave the same molecular formula as that of **4**. Comparing the NMR data of **5** (Tables S2 and S3) with those of **2**, it was found that the only difference was that the acetyl group at C-4″ was missing in **5**, a result which was inferred from the ^1^H-^1^H COSY correlations (Figure 4) of H-1″ (*δ*_H_ 5.87)/H-2″ (*δ*_H_ 3.78)/H-3″ (*δ*_H_ 5.17)/H-4″ (*δ*_H_ 3.79)/H-5″ (*δ*_H_ 3.97), and the HMBC correlation from H-3″ to 3″-COCH_3_ (*δ*_C_ 21.0). Accordingly, compound **5** was determined to be kaempferol 3-*O*-*α*-L-rhamnosyl-(1 → 2)-(3″-acetyl-*β*-D-glucuronate methyl ester).

The structures of the known compounds (**6**–**22**) were identified by comparison of the MS and NMR data with those reported previously, including yakuchinone A (**6**) [34], hexahydrocurcumin (**7**) [35], gingerenone A (**8**) [35], kaempferol 3-*O*-*α*-L-rhamnosyl-(1 → 2)-(*β*-D-glucuronate methyl ester) (**9**) [36], kaempferol 3-*O*-*β*-D-glucoside (**10**) [37], isohamnetin 3-*O*-*β*-glucopyranoside (**11**) [38], 3,5-dihydroxy-4′,7-dimethoxyflavone (**12**) [39], tectochrysin (**13**) [40], pinocembrin (**14**) [41], isoepiphyllocoumarin (**15**) [42], epiphyllocoumarin (**16**) [42], benzyl *O*-*β*-D-glucopyranoside (**17**) [43], (*S*)-1-phenylethyl-*β*-D-glucopyranoside (**18**) [44], 4-hydroxy-2,6-dimethoxyphenyl-*β*-D-glucoside (**19**) [45], 1,4-dihydroxyl-3-methoxyphenyl-4-*O*-*β*-D-glucopyranoside (**20**) [46], pyrocatechol 1-*O-β*-D-glucopyranoside (**21**) [47], and 4,4′-dimethoxy-3′-hydroxy-7,9′:7′,9-diepoxylignan-3-*O*-*β*-D-glucopyranoside (**22**) [48] (Figure 2).

### 3.3. Tyrosinase Inhibitory Activities of Compounds Isolated from A. oxyphylla Leaves and Stems

Tyrosinase inhibitory activities were assessed in the 21 isolated compounds, and the results are shown in Table 1. It can be seen that most of the compounds from *A. oxyphylla* stems and leaves exhibited tyrosinase inhibitory activity; however, different structure skeletons produced different inhibitory effects. Among the active compounds, diarylheptanoid compounds (**1**, **7**, **8**) and lignan glycoside (**22**) exhibited strong inhibitory activity; in particular, the IC_50_ values of compounds **7**, **8**, and **22** were much lower than that of the positive control (kojic acid).

Among the tested compounds, **6**, **7**, and **8** were identified as diarylheptanoid. However, the inhibitory effects of **7** and **8** were much stronger than those of **6**. An analysis of the structure–bioactivity relationships revealed that substituents at the aliphatic chain between the two benzene rings had minimal impact on activity. In contrast, the presence of additional hydroxyl or methoxyl groups at the aromatic ring could markedly enhance bioactivity, potentially due to the polyphenol oxidase activity of tyrosinase. Further analysis of phenolic glycosides **19** and **20** indicated that the absence of a methoxyl group at the C-6 position contributed to increased inhibitory activity. Notably, different superscript values indicate a significant difference (*p* < 0.05) in the same row.

In Table 1, some inconsistencies in the data for inhibition rates and corresponding calculated IC_50_ values may be observed, as with compounds **2** and **6**. These results may have been obtained because the saturation concentration in the tyrosinase reaction arrived before 200 μM, so that the inhibitory rate of 100 μM was close to that of 200 μM; as a result, the IC_50_ value was not what we expected.

### 3.4. DPPH Radical Scavenging Activities of Compounds Isolated from A. oxyphylla Leaves and Stems

The antioxidant activity of 16 isolated compounds was assessed by determination of their DPPH radical scavenging capacity, and the results are shown in Table 2. It can be seen that compounds **12**, **16**, and **20** exhibited certain DPPH radical scavenging capacity at a concentration of 200 μg/mL with inhibitory rates of 76.93–84.06%, with their IC_50_ values between 7.29 and 23.21 μg/mL. In contrast, compounds **1**, **2**, **6**–**9**, **13**–**15**, **17**, **19**, **21**, and **22** all exhibited moderate DPPH radical scavenging capacity, with inhibitory rates of 18.61–60.59% at the same concentration.

### 3.5. ABTS Radical Scavenging Activities of Compounds Isolated from A. oxyphylla Leaves and Stems

The antioxidant activity of **16** isolated compounds was assessed by determination of their ABTS radical scavenging capacity, and the results are shown in Table 3. It can be seen that all the tested compounds except **17** exhibited ABTS radical scavenging capacity. Among them, compounds **1**, **2**, **7**–**9**, **13**, **16**, and **19**–**22** exhibited strong activity, with inhibitory rates of more than 90% at a concentration of 200 μg/mL, and IC_50_ values between 6.42 and 46.03 μg/mL. In contrast, compounds **12**, **14**, and **15** showed some scavenging activity, with inhibitory rates of 71.48–78.11% at the same concentration.

From the above results, we may say that compounds generally isolated from *A. oxyphylla* leaves and stems exhibited a stronger ABTS radical scavenging capacity compared with their DPPH radical scavenging capacity (Table S4). This difference may be attributed to the varying sensitivity of these compounds to different types of free radicals. Previous studies have reported that total flavonoids from the leaves of *A. oxyphylla* possess antioxidant properties [49]. Our results further demonstrated that, in addition to flavonoids, other compounds such as diarylheptanoids and phenolic acids also contribute significantly to the antioxidant capacity of *A. oxyphylla* leaves and stems.

### 3.6. Molecular Orbital Analysis

**Figure 6:**
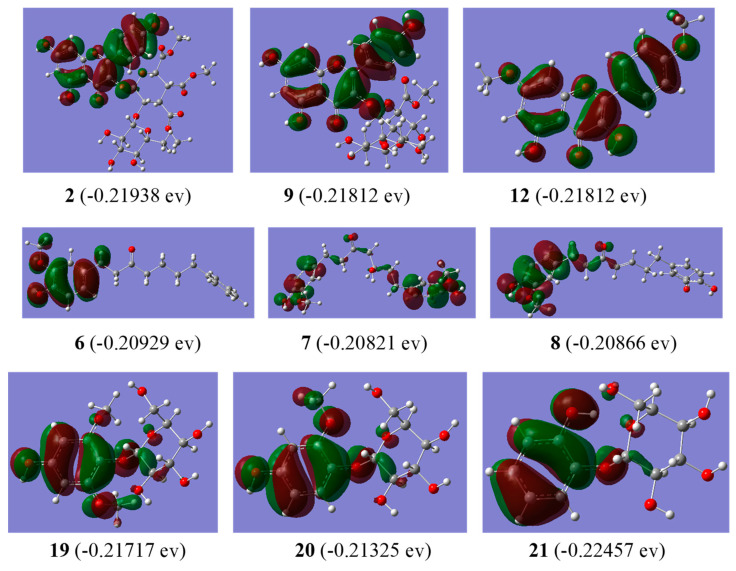
HOMO distribution of compounds **2**, **9**, **12**, **6**–**8**, and **19**–**21** in ethanol.

The HOMO orbital energy is closely associated with electron-donating abilities and serves as an indicator of free radical scavenging potential. Molecules with higher HOMO energy levels typically exhibit stronger antioxidant activity. For phenolic glycosides, the predicted order of electron-donating capability based on the HOMO energy was found to be **20** > **19** > **21** (Figure 6), a result which aligned with the experimental results. The structural variations among these three compounds may be attributed to the presence of OH and OCH_3_ substituents on the aromatic ring. Specifically, the 6-OCH_3_ substituent appears to reduce antioxidant activity, as evidenced by a comparison of compound **19** (IC_50_ = 114.44 μg/mL = 344.70 μmol/L for DPPH, IC_50_ = 17.50 μg/mL = 52.87 μmol/L for ABTS) and compound **20** (IC_50_ = 23.21 μg/mL = 77.03 μmol/L for DPPH, IC_50_ = 8.20 μg/mL = 27.15 μmol/L for ABTS). Conversely, the 4-OH substituent enhances antioxidant activity, as demonstrated by a comparison of **20** and **21** (IC_50_ ≥ 200 μg/mL for DPPH, IC_50_ = 46.03 μg/mL = 169.23 μmol/L for ABTS). However, the correlation between HOMO energy and antioxidant activity varies, depending on the type of free radical. In flavonoids (e.g., compounds **2**, **9**, and **12**), HOMO energy was consistent with DPPH radical scavenging activity, whereas in diarylheptanoids (e.g., compounds **6**, **7**, and **8**), it was consistent with ABTS radical scavenging activity (Figure 6). These discrepancies may be attributed to the differing effects of substituents and steric hindrance on various free radical reactions. Further computational and experimental studies are necessary to elucidate these influences comprehensively.

### 3.7. B16 Melanin Production Inhibition Activities of Compounds Isolated from A. oxyphylla Leaves and Stems

**Figure 7:**
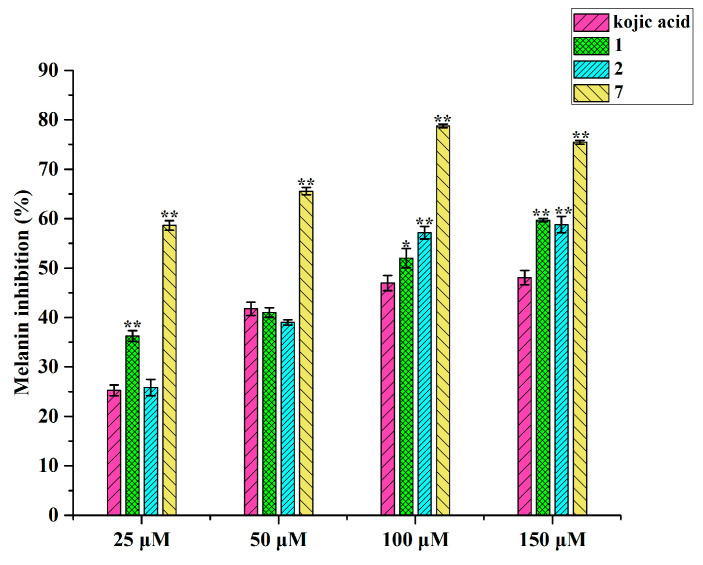
Melanin inhibitory activity of compounds **1**, **2**, and **7** in B16 cells. Each column represents means ± SD of three independent experiments. * *p* < 0.05, ** *p* < 0.01 indicate significant difference from positive control (kojic acid).

Compounds **1**, **2**, and **7** were tested to determine their melanin production inhibition activity in B16 cells, and the results are shown in Figure 7. All three compounds presented no toxicity under the selected concentrations, and all exhibited stronger inhibitory effects than positive control (kojic acid) at a concentration of 150 μM, with the effects presented being concentration dependent.

Compounds **1**, **2**, and **7** demonstrated both tyrosinase inhibitory activity and antioxidant effects, which may explain their ability to inhibit melanin production in B16 cells. At the same time, compounds **1** and **7** were classified as diarylheptanoids, and compound **2** as a flavonoid glycoside, indicating that other analogues within these chemical families may also have similar effects on B16 cells.

## 4. Conclusions

*A. oxyphylla* is a commercially important underwood crop. Its immature fruits are consumed as cold dishes and preserved fruits, while the mature fruits are utilized in traditional Chinese medicine to strengthen the spleen and kidneys, and reduce urine output. However, the leaves and stems of *A. oxyphylla* are usually discarded after 3–4 years of fruit harvesting, indicating an underutilization of these plant parts. In this study, the chemical constituents of ethanol extract from *A. oxyphylla* leaves and stems were systematically separated for the first time. In total, twenty-two compounds were identified, including five new compounds and seventeen known ones. At the same time, nineteen compounds with tyrosinase inhibitory activity and sixteen compounds with DPPH and ABTS radical scavenging activities, as well as three compounds with a melanin production inhibitory effect in B16 cells, were detected. These findings suggest that the diarylheptanoids, flavonoids, phenolic glycosides, and lignan glucosides in *A. oxyphylla* leaves and stems possess significant tyrosinase inhibitory and antioxidant activities, along with potential melanin production inhibition properties. Such functions suggest that *A. oxyphylla* leaves and stems hold promise for application in functional cosmetics, as well in the development of health-promoting food products, due to their rich antioxidant content.

## Figures and Tables

**Table 1:** Tyrosinase inhibitory activity of isolated compounds.

Comp.	Inhibitory Rate (%) ^a^	IC_50_ ± SD (μM)	Comp.	Inhibitory Rate (%) ^a^	IC_50_ ± SD (μM)
**1**	75.38 ± 2.12 **	22.04 ± 1.09 **	**13**	-	
**2**	55.83 ± 3.97 **	103.55 ± 1.43 **	**14**	30.06 ± 4.32 **	
**3**	48.79 ± 3.71 **		**15**	47.26 ± 1.72 **	
**4**	47.59 ± 1.70 **		**16**	-	
**5**	47.59 ± 4.26 **		**17**	14.32 ± 2.58 **	
**6**	59.84 ± 4.78 **	69.26 ± 3.38 **	**18**	10.66 ± 3.90 **	
**7**	88.84 ± 1.06 **	6.26 ± 0.42 **	**19**	17.57 ± 3.99 **	
**8**	72.32 ± 2.12 **	6.80 ± 1.21 **	**20**	60.78 ± 0.80 **	83.34 ± 3.35 **
**9**	54.79 ± 2.11 **	112.58 ± 3.56 **	**21**	21.89 ± 3.03 **	
**10**	51.49 ± 0.90 **	219.31 ± 2.75 **	**22**	82.72 ± 1.06 **	8.86 ± 0.17 **
**12**	26.34 ± 4.86 **		Kojic acid ^b^	99.56 ± 1.15	37.22 ± 1.64

Note: ^a^ the concentration was 200 μM; ^b^ positive control; “-” not active. ** *p* < 0.01 indicates significant difference from the positive control (kojic acid).

**Table 2:** Antioxidant activity of isolated compounds with DPPH method.

Comp.	Inhibitory Rate (%) ^a^	IC_50_ ± SD (μg/mL)	Comp.	Inhibitory Rate (%) ^a^	IC_50_ ± SD (μg/mL)
**1**	50.74 ± 2.31 **		**15**	36.54 ± 3.60 **	
**2**	22.77 ± 1.85 **		**16**	76.93 ± 1.34 **	7.29 ± 0.60 *
**6**	18.77 ± 1.28 **		**17**	25.74 ± 1.49 **	
**7**	35.80 ± 0.77 **		**19**	60.59 ± 0.17 **	114.44 ± 0.14 **
**8**	52.84 ± 2.47 **		**20**	84.06 ± 1.79	23.21 ± 2.88 **
**9**	43.07 ± 3.99 **		**21**	31.39 ± 3.99 **	
**12**	79.80 ± 2.02 **	23.15 ± 3.24 **	**22**	36.91 ± 2.04 **	
**13**	18.61 ± 0.62 **		Vitamin C ^b^	85.44 ± 0.34	6.20 ± 0.41
**14**	27.53 ± 1.96 **				

Note: ^a^ The concentration was 200 μM; ^b^ positive control. * *p* < 0.05, ** *p* < 0.01 indicate significant difference from the positive control (vitamin C).

**Table 3:** Antioxidant activity of isolated compounds with ABTS radical scavenging method.

Comp.	Inhibitory Rate (%) ^a^	IC_50_ ± SD (μg/mL)	Comp.	Inhibitory Rate (%) ^a^	IC_50_ ± SD (μg/mL)
**1**	91.09 ± 0.55 **	39.59 ± 0.53 *	**15**	71.48 ± 0.32 **	30.57 ± 0.93
**2**	93.04 ± 0.10	6.42 ± 0.12 **	**16**	92.51 ± 0.27	8.49 ± 0.06 **
**6**	4.42 ± 0.12 **		**17**	-	
**7**	91.09 ± 0.41 **	13.17 ± 0.18 **	**19**	92.57 ± 0.10	17.50 ± 0.23 **
**8**	90.81 ± 0.24 **	36.06 ± 1.07 *	**20**	92.80 ± 0.31	8.20 ± 0.14 **
**9**	92.39 ± 0.27	6.20 ± 0.05 **	**21**	92.57 ± 0.27	46.03 ± 0.27 **
**12**	75.07 ± 0.46 **	8.55 ± 0.08 **	**22**	91.51 ± 0.21 **	33.32 ± 0.29
**13**	91.93 ± 1.68 **	20.81 ± 0.29 **	trolox ^b^	92.69 ± 0.03	31.61 ± 0.60
**14**	78.11 ± 0.32 **	27.73 ± 0.69 *			

Note: ^a^ The concentration was 200 μM; ^b^ positive control; “-” not active. * *p* < 0.05, ** *p* < 0.01 indicate significant difference from the positive control (trolox).

## Data Availability

All of the data are included in the article/Supplementary Materials.

## Supplementary Materials

The following supporting information can be downloaded at: https://www.mdpi.com/article/10.3390/antiox13121538/s1: Table S1: ^1^H (500 MHz) and ^13^C NMR (125 MHz) spectral data of compound **1** (MeOD); Table S2: ^1^H NMR spectral data of compounds **2**–**5** (500 MHz, MeOD, *J* in Hz); Table S3: ^13^C NMR spectral data of compounds **2**–**5** (125 MHz, MeOD); Table S4: The inhibitory rate of the compounds for tyrosinase inhibitory activity, and DPPH and ABTS radical scavenging activity; Figure S1. HRESIMS spectrum of compound **1**; Figure S2. ^1^H NMR spectrum of compound **1** in CD_3_OD; Figure S3. ^13^C NMR spectrum of compound **1** in CD_3_OD; Figure S4. HSQC spectrum of compound **1** in CD_3_OD; Figure S5. ^1^H-^1^H COSY spectrum of compound **1** in CD_3_OD; Figure S6. HMBC spectrum of compound **1** in CD_3_OD; Figure S7. ROESY spectrum of compound **1** in CD_3_OD; Figure S8. HRESIMS spectrum of compound **2**; Figure S9. ^1^H NMR spectrum of compound **2** in CD_3_OD; Figure S10. ^13^C NMR spectrum of compound **2** in CD_3_OD; Figure S11. HSQC spectrum of compound **2** in CD_3_OD; Figure S12. ^1^H-^1^H COSY spectrum of compound **2** in CD_3_OD; Figure S13. HMBC spectrum of compound **2** in CD_3_OD; Figure S14. ROESY spectrum of compound **2** in CD_3_OD; Figure S15. HRESIMS spectrum of compound **3;** Figure S16. ^1^H NMR spectrum of compound **3** in CD_3_OD; Figure S17. ^13^C NMR spectrum of compound **3** in CD_3_OD; Figure S18. HSQC spectrum of compound **3** in CD_3_OD; Figure S19. ^1^H-^1^H COSY spectrum of compound **3** in CD_3_OD; Figure S20. HMBC spectrum of compound **3** in CD_3_OD; Figure S21. ROESY spectrum of compound **3** in CD_3_OD; Figure S22. HRESIMS spectrum of compound **4**; Figure S23. ^1^H NMR spectrum of compound **4** in CD_3_OD; Figure S24. ^13^C NMR spectrum of compound **4** in CD_3_OD; Figure S25. HSQC spectrum of compound **4** in CD_3_OD; Figure S26. ^1^H-^1^H COSY spectrum of compound **4** in CD_3_OD; Figure S27. HMBC spectrum of compound **4** in CD_3_OD; Figure S28. ROESY spectrum of compound **4** in CD_3_OD; Figure S29. HRESIMS spectrum of compound **5**; Figure S30. ^1^H NMR spectrum of compound **5** in CD_3_OD; Figure S31. ^13^C NMR spectrum of compound **5** in CD_3_OD; Figure S32. HSQC spectrum of compound **5** in CD_3_OD; Figure S33. ^1^H-^1^H COSY spectrum of compound **5** in CD_3_OD; Figure S34. HMBC spectrum of compound **5** in CD_3_OD; Figure S35. ROESY spectrum of compound **5** in CD_3_OD; Computational details.

## References

[1] Feng D., Fang Z., Zhang P. (2022). The melanin inhibitory effect of plants and phytochemicals: A systematic review. Phytomedicine.

[2] Briganti S., Camera E., Picardo M. (2003). Chemical and instrumental approaches to treat hyperpigmentation. Pigment Cell Res..

[3] Hsu J.-Y., Liu H.-H., Lin T.-H., Li T.-S., Tseng C.-Y., Wong Y., Chen J.-H. (2020). Anti-melanogenesis effects of lotus seedpod in vitro and in vivo. Nutrients.

[4] Park H.Y., Kosmadaki M., Yaar M., Gilchrest B.A. (2009). Cellular mechanisms regulating human melanogenesis. Cell. Mol. Life Sci..

[5] Hwang J.-H., Lee B.M. (2007). Inhibitory effects of plant extracts on tyrosinase L-DOPA oxidation, and melanin synthesis. J. Toxicol. Environ. Health A.

[6] Wang S., Liu X.-M., Zhang J., Zhang Y.-Q. (2014). An efficient preparation of mulberroside A from the branch bark of mulberry and its effect on the inhibition of tyrosinase activity. PLoS ONE.

[7] Lee H.-S. (2002). Tyrosinase inhibitors of *Pulsatilla cernua* root-derived materials. J. Agric. Food Chem..

[8] Lan W.-C., Tzeng C.-W., Lin C.-C., Yen F.-L., Ko H.-H. (2013). Prenylated flavonoids from *Artocarpus altilis*: Antioxidant activities and inhibitory effects on melanin production. Phytochemistry.

[9] Park D.H., Lee J.W., Jin Q., Jeon W.K., Hwang B.Y. (2014). A new noreudesmane-type sesquiterpenoid from *Alpinia oxyphylla*. Bull. Korean Chem. Soc..

[10] Xu J.J., Tan N.H., Xiong J., Adebayo A.H., Han H.J., Zeng G.Z., Ji C.J., Zhang Y.M., Zhu M.J. (2009). Oxyphyllones A and B, novel sesquiterpenes with an unusual 4,5-secoeudesman skeleton from *Alpinia oxyphylla*. Chin. Chem. Lett..

[11] Qiu C.X., Mu L.P., Wang J., Tang R., Hou B., Hu W., Zhang R., Chen X. (2023). Sesquiterpenoids from the fruits of *Alpinia oxyphylla* Miq. and their neuroprotective effect. Phytochemistry.

[12] Bian Q.Y., Wang S.Y., Xu L.J., Chan C.O., Mok D.K.W., Chen S.B. (2013). Two new antioxidant diarylheptanoids from the fruits of *Alpinia oxyphylla*. J. Asian Nat. Prod. Res..

[13] Song W.J., Li Y.H., Wang J.G., Li Z.Y., Zhang J.Q. (2014). Characterization of nucleobases and nucleosides in the fruit of *Alpinia oxyphylla* cellected from different cultivation regions. Drug. Test. Anal..

[14] Yu S.H., Kim H.J., Jeon S.Y., Kim M.R., Lee B.S., Kim D.S., Lee Y.C. (2020). Anti-inflammatory and anti-nociceptive activities of *Alpinia oxyphylla* Miquel extracts in animal models. J. Ethnopharmacol..

[15] Yuan Y., Tan Y.F., Xu P., Li H., Li Y., Chen W., Zhang J., Chen F., Huang G. (2014). Izalpinin from fruits of *Alpinia oxyphylla* with antagonistic activity against the rat bladder contractility. Afr. J. Tradit. Complement. Altern. Med..

[16] Ying L., Wang D., Du G. (2021). Analysis of bioactive components in the fruit, roots, and leaves of *Alpinia oxyphylla* by UPLC-MS/MS. Evid.-Based Complement. Altern. Med..

[17] Li J.T., Zhao Y.H., Lv Y., Su X., Mei W.L., Lu Y.P., Zheng P.H., Zhang Z.L., Zhang X.X., Chen H.Q. (2023). Evaluating the antioxidant properties of the leaves and stems of *Alpinia oxyphylla* in vitro and its growth-promoting, muscle composition change, and antioxidative stress function on juvenile *Litopenaeus vannamei*. Antioxidants.

[18] Hu H.B., Liang H.P., Li H.M., Yuan R.N., Sun J., Zhang L.L., Han M.H., Wu Y. (2018). Isolation, purification, characterization and antioxidant activity of polysaccharides from the stem barks of *Acanthopanax leucorrhizus*. Carbohyd. Polym..

[19] Payet B., Sing A.S.C., Smadja J. (2005). Assessment of antioxidant activity of cane brown sugars by ABTS and DPPH radical scavenging assays: Determination of their polyphenolic and volatile constituents. J. Agric. Food Chem..

[20] Frisch M.J., Schlegel H.B., Scuseria G.E., Robb M.A., Cheeseman J.R., Scalmani G., Barone V., Petersson G.A., Nakatsuji H., Li X. (2016). Gaussian 16.

[21] Rappoport D., Furche F. (2010). Property-optimized Gaussian basis sets for molecular response calculations. J. Chem. Phys..

[22] Lee C., Yang W., Parr R.G. (1988). Development of the Colle-Salvetti correlation-energy formula into a functional of the electron density. Phys. Rev. B.

[23] Miehlich B., Savin A., Stoll H., Preuss H. (1989). Results obtained with the correlation energy density functionals of becke and Lee, Yang and Parr. Chem. Phys. Lett..

[24] Weigend F. (2006). Accurate Coulomb-fitting basis sets for H to Rn. Phys. Chem. Chem. Phys..

[25] Zhao Y., Truhlar D.G. (2008). The M06 suite of density functionals for main group thermochemistry, thermochemical kinetics, noncovalent interactions, excited states, and transition elements: Two new functionals and systematic testing of four M06-class functionals and 12 other functionals. Theor. Chem. Acc..

[26] Grimme S., Ehrlich S., Goerigk L. (2011). Effect of the damping function in dispersion corrected density functional theory. J. Comput. Chem..

[27] Grimme S., Antony J., Ehrlich S., Krieg H. (2010). A consistent and accurate ab initio parametrization of density functional dispersion correction (DFT-D) for the 94 elements H-Pu. J. Chem. Phys..

[28] Marenich A.V., Cramer C.J., Truhlar D.G. (2009). Universal solvation model based on solute electron density and on a continuum model of the solvent defined by the bulk dielectric constant and atomic surface tensions. J. Phys. Chem. B.

[29] Xie J., Li M.-X., Du Z.-Z. (2022). Chemical compounds, anti-aging and antibacterial properties of *Rosa rugosa* Purple branch. Ind. Crops Prod..

[30] Mosmann T.J. (1983). Rapid colorimetic assay for cellular growth and surfival: Application to proliferation and cytotoxicity assays. J. Immunol. Methods.

[31] Li G., Cui J.-M., Kwon Y., Seo C.-S., Lee C.-S., Woo M.-H., Lee E.S., Jahng Y., Chang H.-W., Lee S.-H. (2005). Two new diarylheptanoids from *Juglans mandshurica*. Bull. Korean Chem. Soc..

[32] Simon A., Chulia A.J., Kaouadji M., Allais D.P., Delage C. (1993). Two flavonol 3-[triacetylarabinostyl (1-6) glycosides] from *Calluna vulgaris*. Phytochemistry.

[33] Shahat A.A., Apers S., Miert S.V., Claeys M., Pieters L., Vlietinck A. (2001). Structure elucidation of three new acetylated flavonoid glycosides from *Centaurium spicatum*. Magn. Reson. Chem..

[34] Miyazawa M., Nakamura Y., Sakano K., Nakamura S.-I., Kosaka H. (2001). Suppression of SOS-inducing activity of chemical mutagens by yakuchinone A from *Alpinia oxyphylla* in the *Salmonella typhimurium* TA1535/sSK1002 umu test. J. Oleo Sci..

[35] Hori Y., Miura T., Hirai Y., Fukumura M., Nemoto Y., Toriizuka K., Ida Y. (2003). Pharmacognostic studies on ginger and related drugs-Part 1: Five sulfonated compounds from *Zingiberis rhizome* (Shokyo). Phytochemistry.

[36] Tram N.C.T., Son N.T., Nga N.T., Phuong V.T.T., Cue N.T., Phuong D.T., Truan G., Cuong N.M., Thao D.T. (2017). The hepatoptotective activity of a new derivative kaempferol glycoside from the leaves of Vietnamese *Phyllanthus acidus* (L.) skeels. Med. Chem. Res..

[37] Markham K.R., Ternal B., Stanley R., Geiger H., Mabry T.J. (1978). Carbon-13 NMR studies of flavonoids-III. Naturally occuring flavonoid glycosides and their acylated derivtives. Tetrahedron.

[38] Beck M.-A., Haberlein H. (1999). Flavonol glycosides from *Eschscholtzia californica*. Phytochemistry.

[39] Gadallah A.S., Mujeeb-ur-Rehman, Atta-ur-Rahman, Yousuf S., Atia-tul-Wahab, Jabeen A., Swilam M.M., Khalifa S.A.M., EI-Seedi H.R., Choudhary M.I. (2020). Anti-inflammatory principles from *Tamarix aphylla* L.: A bioassay-guided fractionation study. Molecules.

[40] Xu J., Tan N., Zeng G., Han H., Huang H., Ji C., Zhu M., Zhang Y. (2009). Studies on chemical constituents in fruits of *Alpinia oxyphylla*. China J. Chin. Mater. Med..

[41] Jung J.H., Pummangura S., Chaichantipyuth C., Patarapanich C., Mclaughlin J.L. (1990). Bioactive constituents of *Melodorum fruticosum*. Phytochemistry.

[42] Tang W.-J., Zhang Y.-L., Xiao Q.-P., Huang C., Jin Y., Li J. (2013). Four flavanocoumarins from the leaves of *Litsea coreana* Levl. Chem. Biodivers..

[43] Marino S.D., Festa C., Zollo F., Iorizzi M. (2009). Phenolic glycosides from *Cucumis melo* var. inodorus seeds. Phytochem. Lett..

[44] Xie B.-B., Hou L., Guo B.-L., Huang W.-H., Yu J.-G. (2014). The compounds from n-butanol fraction of *Alpinia oxyphylla*. Acta Pharm. Sin..

[45] Zhao Q.-C., Li Y., Cai H.-M., Guo T., Wu L.-J. (2009). Chemical constituents of *Clerodendron cyrtophyllum* Turcz. (II). Chin. J. Med. Chem..

[46] Wu Z.-J., Ouyang M.-A., Wang S.-B. (2008). Two new phenolic water-soluble constituents from branch bark of *Davidia involucrata*. Nat. Prod. Res..

[47] Itoh A., Tanahashi T., Ikejima S., Inoue M., Nagakura N., Inoue K., Kuwajima H., Wu H.-X. (2000). Five phenolic glycosides from *Alangium Chinese*. J. Nat. Prod..

[48] Li N., Tan N.-H., Zhou J. (2003). A new lignan glycoside from *Curculigo capitulata*. Acta Bot. Yunnanica.

[49] Niu Q., Gao Y., Liu P. (2020). Optimization of microwave-assisted extraction, antioxidant capacity, and characterization of total flavonoids from the leaves of *Alpinia oxyphylla* Miq. Prep. Biochem. Biotechnol..

