# Supplementary material for: Identification of Chemical Constituents from Leaves and Stems of Alpinia oxyphylla: Potential Antioxidant and Tyrosinase Inhibitory Properties
Source: Antioxidants (Basel). 2024 Dec 16;13(12):1538. doi: 10.3390/antiox13121538 (PMC11727410; doi:10.3390/antiox13121538)
Supplement: Supplementary file 1 [file antioxidants-13-01538-s001.zip › antioxidants-3281633-supplementary.pdf]

Supplementary material

Identification of Chemical Constituents from Leaves and Stems of *Alpinia oxyphylla*: Potential Antioxidant and Tyrosinase Inhibitory Properties

Hui-Qin Chen[1,2,†], Xin Su[1,2,†], Pan Xiang[3], Yan-Mei Wei[1,2], Hao Wang[1,2], Jun-Tao Li[1,2], Shou-Bai Liu[4], Wen-Li Mei[1,2,*], and Hao-Fu Dai[1,2,*]

[1] *Hainan Key Laboratory of Research and Development of Natural Product from Li Folk Medicine, Institute of Tropical Bioscience and Biotechnology, Chinese Academy of Tropical Agricultural Sciences, Haikou 571101, P.R. China*

[2] *Hainan Institute for Tropical Agricultural Resources, Haikou 571101, P.R. China.*

[3] *Zhongshan Institute for Drug Discovery, Shanghai Institute of Materia Medica, Chinese Academy of Sciences, Zhongshan 528400, P.R. China*

[4] *Key Laboratory of Genetics and Germplasm Enhancement in Tropical Specific Forest Trees and Ornamental Plants, Ministry of Education/Hainan Key Laboratory for Biology of Tropical Specific Ornamental Plants Germplasm, School of Tropical Agriculture and Forestry, Hainan University, Haikou 570228, P. R. China*

[†] These authors contributed equally for this work.

***Corresponding authors: Prof. Dr. Wen-Li Mei,** E-mail: meiwenli@itbb.org.cn

Prof. Dr. Hao-Fu Dai, E-mail: daihaofu@itbb.org.cn

Contents

Table S1 ^{1}H (500 MHz) and ^{13}C NMR (125 MHz) spectral data of compound **1** (MeOD) ………..3
Table S2 ^{1}H NMR spectral data of compounds **2 - 5** (500 MHz, MeOD, *J* in Hz) ………………..4
Table S3 ^{13}C NMR spectral data of compounds **2 - 5** (125 MHz, MeOD) ………………………..5
Table S4 The inhibitory rate of the compounds for tyrosinase inhibitory activity, and DPPH and ABTS radical scavenging activity……………………………………………..………………6
Figure S1. HRESIMS spectrum of compound **1**…………………………………………………..7
Figure S2. ^{1}H NMR spectrum of compound **1** in CD_3OD………………………………………..7
Figure S3. ^{13}C NMR spectrum of compound **1** in CD_3OD………………………………………..8
Figure S4. HSQC spectrum of compound **1** in CD_3OD……………………………………………8
Figure S5. ^{1}H-^{1}H COSY spectrum of compound **1** in CD_3OD……………………………………9
Figure S6. HMBC spectrum of compound **1** in CD_3OD…………………………………………9
Figure S7. ROESY spectrum of compound **1** in CD_3OD…………………………………………10
Figure S8. HRESIMS spectrum of compound **2** …………………………………………………10
Figure S9. ^{1}H NMR spectrum of compound **2** in CD_3OD………………………………………11
Figure S10. ^{13}C NMR spectrum of compound **2** in CD_3OD……………………………………11
Figure S11. HSQC spectrum of compound **2** in CD_3OD…………………………………………12
Figure S12. ^{1}H-^{1}H COSY spectrum of compound **2** in CD_3OD…………………………………12
Figure S13. HMBC spectrum of compound **2** in CD_3OD ………………………………………13
Figure S14. ROESY spectrum of compound **2** in CD_3OD………………………………………13
Figure S15. HRESIMS spectrum of compound **3** ……………………………………………..14
Figure S16. ^{1}H NMR spectrum of compound **3** in CD_3OD…………………………………..14
Figure S17. ^{13}C NMR spectrum of compound **3** in CD_3OD…………………………………..15
Figure S18. HSQC spectrum of compound **3** in CD_3OD……………………………………..15
Figure S19. ^{1}H-^{1}H COSY spectrum of compound **3** in CD_3OD………………………………..16
Figure S20. HMBC spectrum of compound **3** in CD_3OD ……………………………………..16
Figure S21. ROESY spectrum of compound **3** in CD_3OD………………………………………..17
Figure S22. HRESIMS spectrum of compound **4** ………………………………………………..17
Figure S23. ^{1}H NMR spectrum of compound **4** in CD_3OD……………………………………..18
Figure S24. ^{13}C NMR spectrum of compound **4** in CD_3OD……………………………………..18
Figure S25. HSQC spectrum of compound **4** in CD_3OD………………………………………..19
Figure S26. ^{1}H-^{1}H COSY spectrum of compound **4** in CD_3OD………………………………..19
Figure S27. HMBC spectrum of compound **4** in CD_3OD ……………………………………..20
Figure S28. ROESY spectrum of compound **4** in CD_3OD……………………………………..20
Figure S29. HRESIMS spectrum of compound **5** ………………………………………………..21
Figure S30. ^{1}H NMR spectrum of compound **5** in CD_3OD……………………………………..21
Figure S31. ^{13}C NMR spectrum of compound **5** in CD_3OD……………………………………..22
Figure S32. HSQC spectrum of compound **5** in CD_3OD………………………………………..22
Figure S33. ^{1}H-^{1}H COSY spectrum of compound **5** in CD_3OD…………………………………..23
Figure S34. HMBC spectrum of compound **5** in CD_3OD ……………………………………….23
Figure S35. ROESY spectrum of compound **5** in CD_3OD………………………………………..24
Computational details…………………………………………………………………………..25

Table S1 ^{1}H (500 MHz) and ^{13}C NMR (125 MHz) spectral data of compound **1** (MeOD)

Position	δ_H (*J* in Hz)	δ_C, type
1, 1''	5.12, d (6.0)	81.5, CH
2, 2''	3.33, m, overlapped	58.6, CH
3, 3''	4.73, td (7.0, 2.9)	79.9, CH
4, 4''	2.05, dt (13.9, 8.0)	36.6, CH_2
	1.82, ddd (13.9, 8.0, 4.0)	
5, 5''	3.26, m	72.7, CH
6, 6''	3.47, m	76.1, CH
7, 7''	2.55, m	40.5, CH_2
	2.51, m	
8, 8''		132.1, C
9, 9''	6.70, d (1.8)	114.0, CH
10, 10''		148.7, C
11, 11''		145.7, C
12, 12''	6.65, d (8.0)	116.0, CH
13, 13''	6.54, dd (8.0, 1.8)	122.8, CH
1', 1'''		135.0, C
2', 2'''	6.69, s	105.4, CH
3', 3'''		149.4, C
4', 4'''		136.3, C
5', 5'''		149.4, C
6', 6'''	6.69, s	105.4, CH
10, 10''-OCH_3	3.76, s	56.3, CH_3
3', 5', 3''', 5'''-OCH_3	3.80, s	56.8, CH_3

Table S2 ^{1}H NMR spectral data of compounds **2 - 5** (500 MHz, MeOD, *J* in Hz)

Position	**2**	**3**	**4**	**5**
6	6.17, d (2.1)	6.19, d (2.1)	6.20, d (2.1)	6.20, d (2.1)
8	6.37, d (2.1)	6.38, d (2.1)	6.40, d (2.1)	6.39, d (2.1)
2'	8.02, d (8.8)	7.60, d (2.2)	8.05, d (8.9)	8.00, d (8.8)
3'	6.92, d (8.8)		6.94, d (8.9)	6.91, d (8.8)
5'	6.92, d (8.8)	6.90, d (8.4)	6.94, d (8.9)	6.91, d (8.8)
6'	8.02, d (8.8)	7.58, dd (8.4, 2.2)	8.05, d (8.9)	8.00, d (8.8)
1''	5.90, d (7.5)	5.90, d (7.5)	5.83, d (7.6)	5.87, d (7.5)
2''	3.90, t (8.3)	3.97, dd (9.3, 7.5)	3.74, m	3.78, overlapped
3''	5.39, t (9.3)	5.38, t (9.3)	3.88, t (9.3)	5.17, t (9.3)
4''	5.08, t (9.7)	5.13, t (9.8)	4.90, d (9.8)	3.79, d (4.9)
5''	4.23, dt (10.9, 3.1)	4.19, d (9.8)	4.05, m	3.94, d (9.9)
3''-CO**CH_3**	2.11, s	2.11, s		2.17, s
4''-CO**CH_3**	1.98, s	1.98, s	2.07, s	
6''-OCH_3	3.58, s	3.58, s	3.59, s	3.65, s
1'''	4.89, overlapped	4.89, overlapped	5.23, d (1.7)	4.87, overlapped
2'''	3.80, dd (5.6, 3.2)	3.77, dd (3.4, 1.6)	4.00, dd (3.4, 1.7)	3.77, overlapped
3'''	3.71, dd (9.8, 3.2)	3.70, dd (9.6, 3.4)	3.78, m	3.71, dd (9.7, 3.4)
4'''	3.34, s	3.34, d (9.6)	3.37, d (9.6)	3.34, d (9.7)
5'''	4.01, m	4.01, m	4.03, d (10.0)	4.00, m
6'''	0.99, d (6.2)	0.98, d (6.2)	0.98, d (6.2)	0.97, d (6.2)

Table S3 ^{13}C NMR spectral data of compounds **2 - 5** (125 MHz, MeOD)

Position	**2**	**3**	**4**	**5**
2	159.1, C	159.0, C	159.2, C	159.0, C
3	134.0, C	134.1, C	134.1, C	134.0, C
4	178.7, C	178.8, C	179.0, C	178.9, C
5	163.1, C	163.2, C	163.2, C	163.2, C
6	99.8, CH	99.8, CH	99.8, CH	99.9, CH
7	165.8, C	165.8, C	165.8, C	165.8, C
8	94.7, CH	94.6, CH	94.7, CH	94.7, CH
9	158.4, C	158.4, C	158.4, C	158.4, C
10	105.9, C	105.9, C	105.9, C	105.9, C
1′	122.8, C	123.1, C	122.9, C	122.8, C
2′	132.2, CH	117.3, CH	132.2, CH	132.1, CH
3′	116.2, CH	146.0, C	116.1, CH	116.2, CH
4′	161.5, C	149.8, C	161.4, C	161.4, C
5′	116.2, CH	116.1, CH	116.1, CH	116.2, CH
6′	132.2, CH	123.3, CH	132.2, CH	132.1, CH
1″	100.1, CH	100.2, CH	100.2, CH	100.4, CH
2″	78.2, CH	78.3, CH	79.3, CH	78.6, CH
3″	75.4, CH	75.5, CH	75.7, CH	77.9, CH
4″	71.1, CH	71.1, CH	73.4, CH	71.2, CH
5″	73.3, CH	73.4, CH	73.7, CH	76.6, CH
6″	169.0, C	169.1, C	169.5, C	170.1, C
3″-**C**OCH_3	171.5, C	171.5, C		172.0, C
3″-CO**CH_3**	20.7, CH_3	20.7, CH_3		21.0, CH_3
4″-**C**OCH_3	171.4, C	171.4, C	171.9, C	
4″-CO**CH_3**	20.5, CH_3	20.5, CH_3	20.7, CH_3	
6″-OCH_3	53.1, CH_3	53.2, OCH_3	53.0, OCH_3	52.9, OCH_3
1‴	103.1, CH	103.1, CH	102.8, CH	103.0, CH
2‴	72.4, CH	72.5, CH	72.3, CH	72.5, CH
3‴	72.1, CH	72.1, CH	72.3, CH	72.2, CH
4‴	73.7, CH	73.7, CH	73.9, CH	73.8, CH
5‴	70.4, CH	70.4, CH	70.0, CH	70.3, CH
6‴	17.5, CH_3	17.5, CH_3	17.5, CH_3	17.5, CH_3

Table S4 The inhibitory rate of the compounds for tyrosinase inhibitory activity, and DPPH and ABTS radical scavenging activity

Comp.	Inhibitory rate (%)		
	Tyrosinase inhibitory	DPPH	ABTS
**1**	75.38 ± 2.12	50.74 ± 2.31	91.09 ± 0.55
**2**	55.83 ± 3.97	22.77 ± 1.85	93.04 ± 0.10
**3**	48.79 ± 3.71		
**4**	47.59 ± 1.70		
**5**	47.59 ± 4.26		
**6**	59.84 ± 4.78	18.77 ± 1.28	4.42 ± 0.12
**7**	88.84 ± 1.06	35.80 ± 0.77	91.09 ± 0.41
**8**	72.32 ± 2.12	52.84 ± 2.47	90.81 ± 0.24
**9**	54.79 ± 2.11	43.07 ± 3.99	92.39 ± 0.27
**10**	51.49 ± 0.90		
**12**	26.34 ± 4.86	79.80 ± 2.02	75.07 ± 0.46
**13**	—	18.61 ± 0.62	91.93 ± 1.68
**14**	30.06 ± 4.32	27.53 ± 1.96	78.11 ± 0.32
**15**	47.26 ± 1.72	36.54 ± 3.60	71.48 ± 0.32
**16**	—	76.93 ± 1.34	92.51 ± 0.27
**17**	14.32 ± 2.58	25.74 ± 1.49	—
**18**	10.66 ± 3.90		
**19**	17.57 ± 3.99	60.59 ± 0.17	92.57 ± 0.10
**20**	60.78 ± 0.80	84.06 ± 1.79	92.80 ± 0.31
**21**	21.89 ± 3.03	31.39 ± 3.99	92.57 ± 0.27
**22**	82.72 ± 1.06	36.91 ± 2.04	91.51 ± 0.21
Kojic acid	99.56 ± 1.15		
Vitamin C		85.44 ± 0.34	
Trolox			92.69 ± 0.03

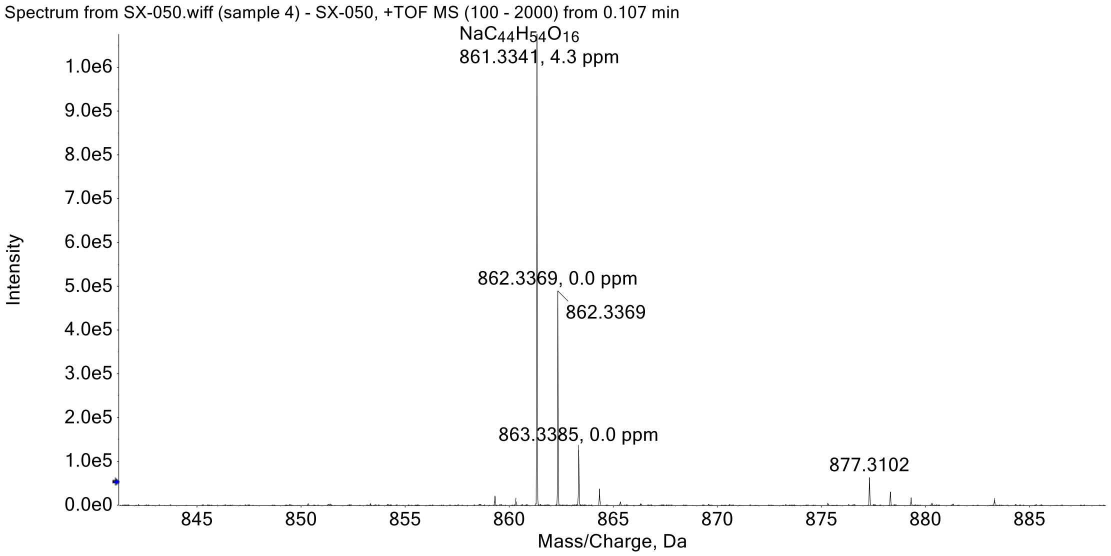

Figure S1. HRESIMS spectrum of compound **1**

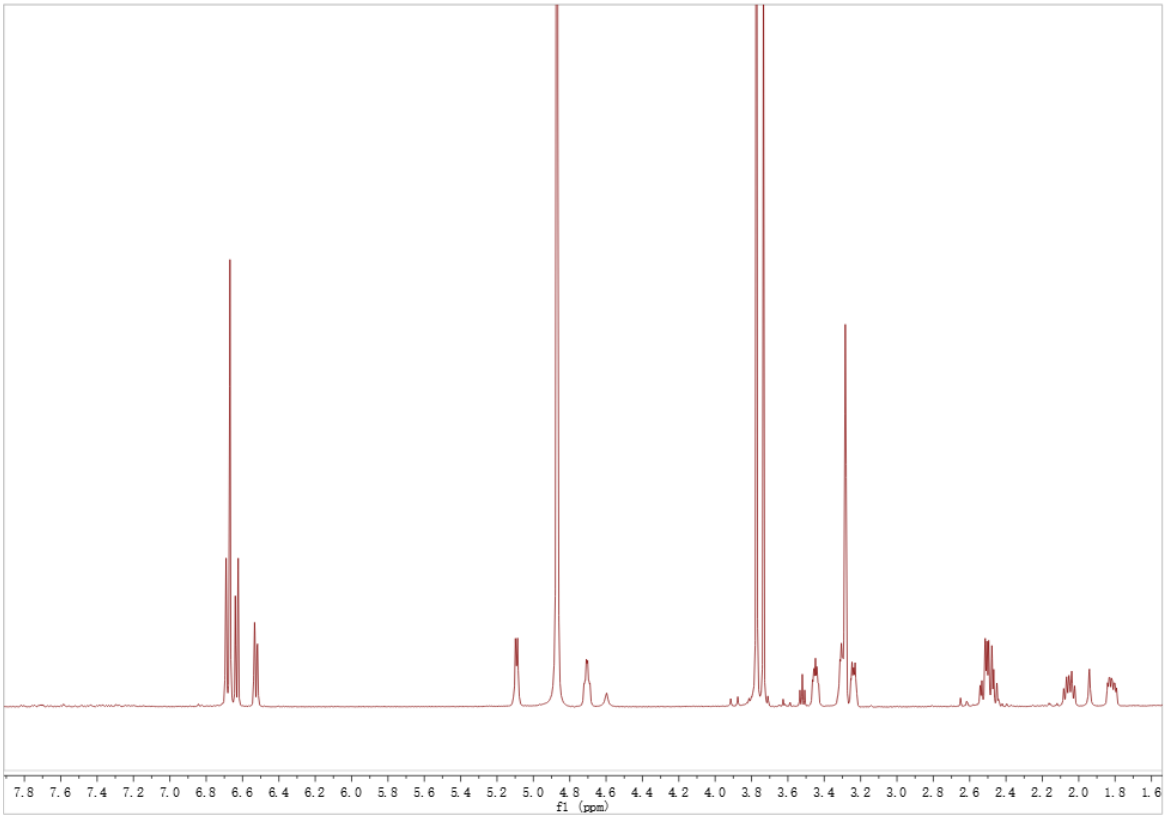

Figure S2. ^{1}H NMR spectrum of compound **1** in CD_3OD

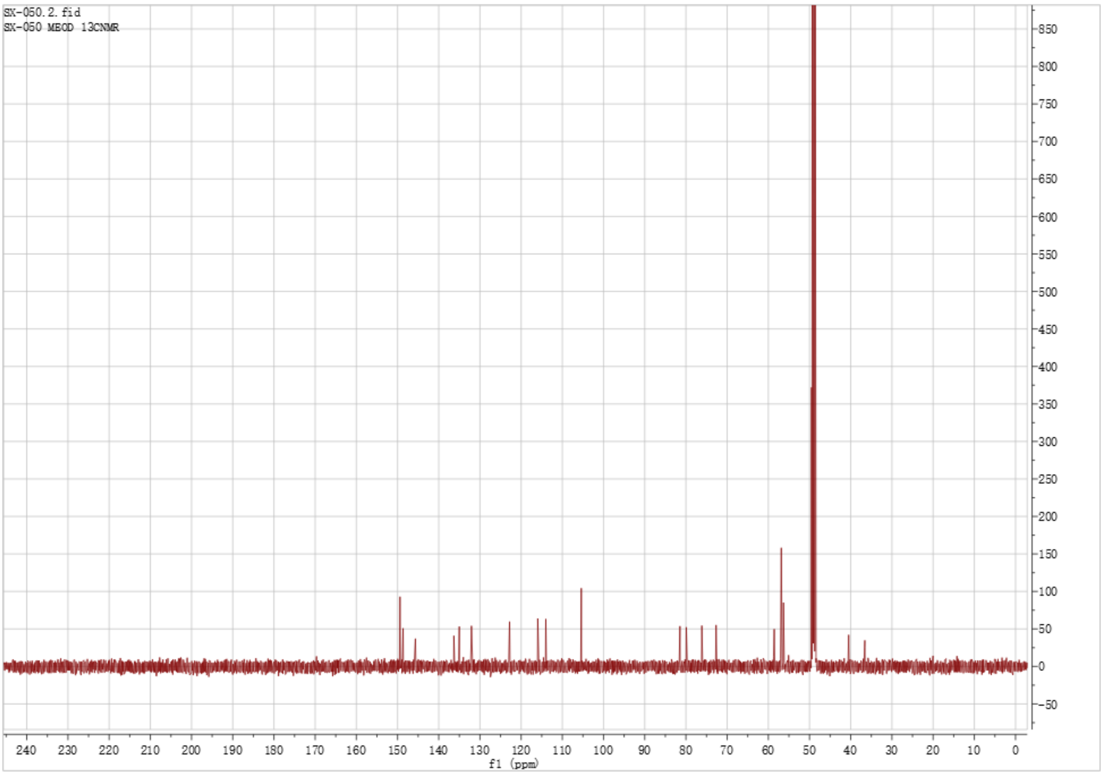

Figure S3. ^{13}C NMR spectrum of compound **1** in CD_3OD

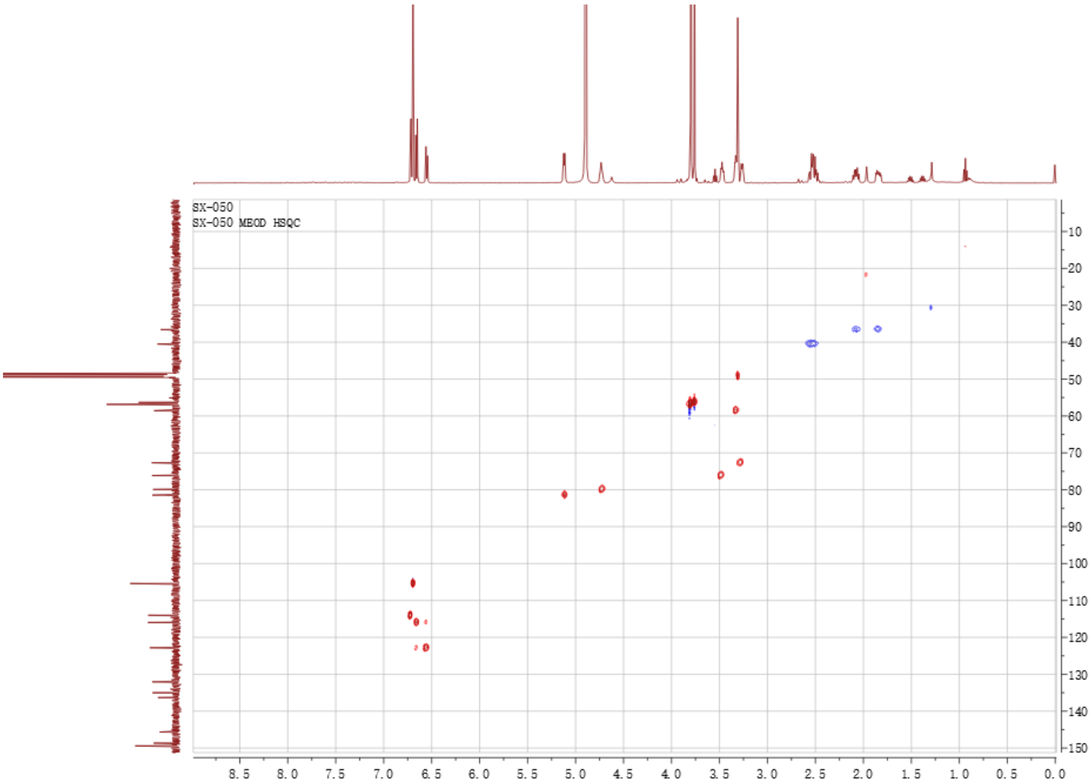

Figure S4. HSQC spectrum of compound **1** in CD_3OD

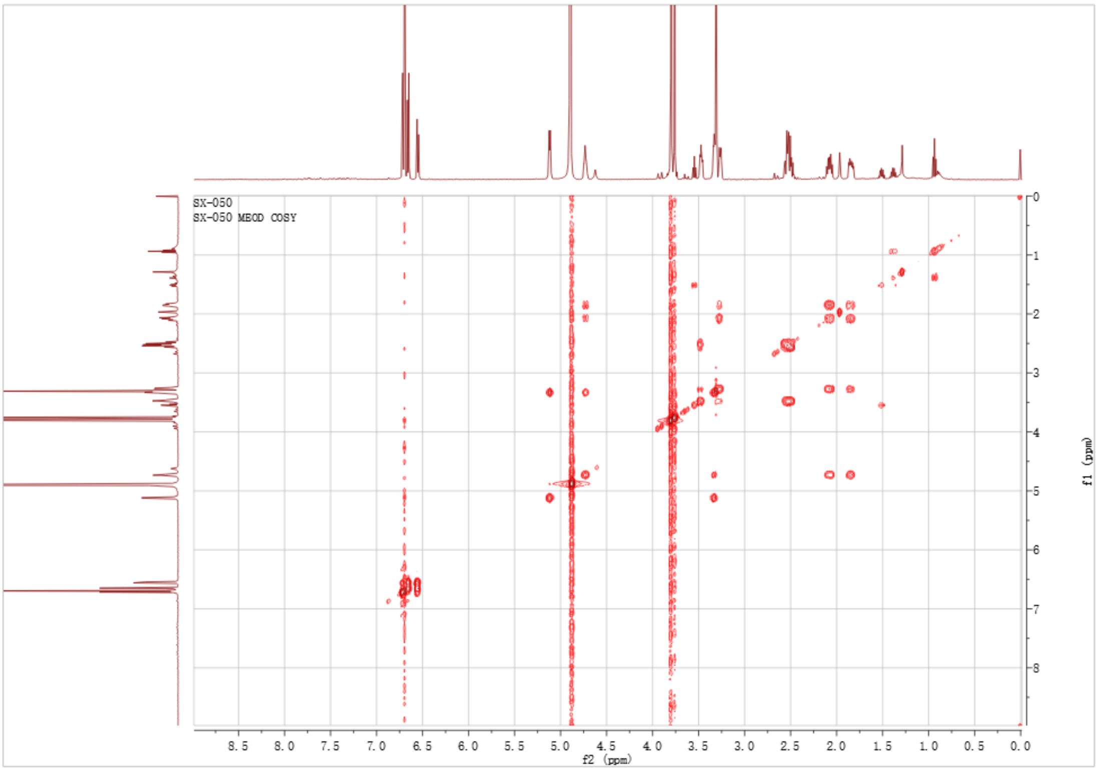

Figure S5. ^{1}H-^{1}H spectrum of compound **1** in CD_3OD

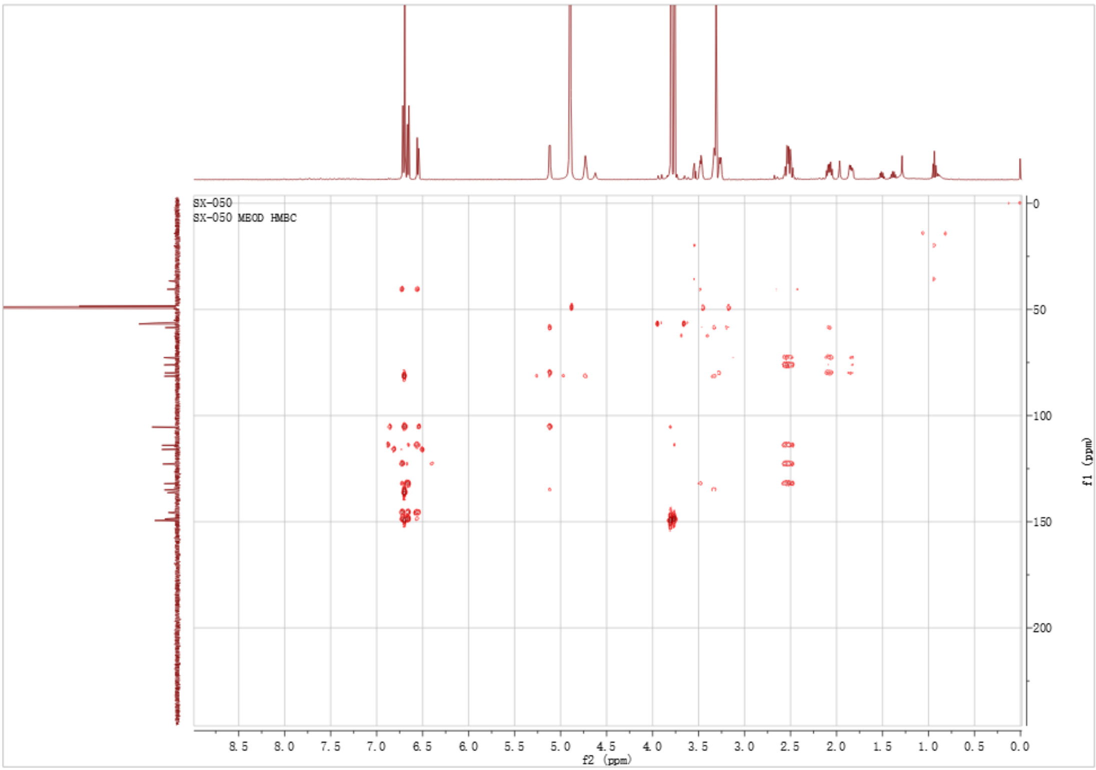

Figure S6. HMBC spectrum of compound **1** in CD_3OD

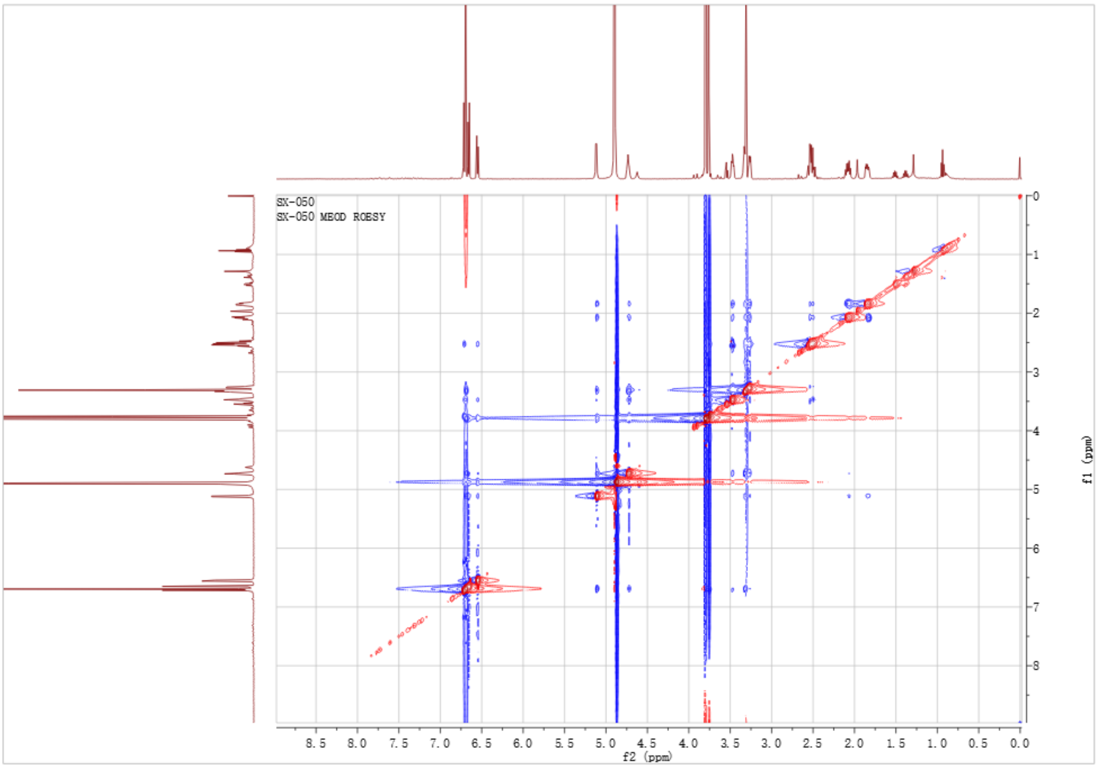

Figure S7. ROESY spectrum of compound **1** in CD_3OD

User Spectra

Fragmentor Voltage	Collision Energy	Ionization Mode
135	0	ESI

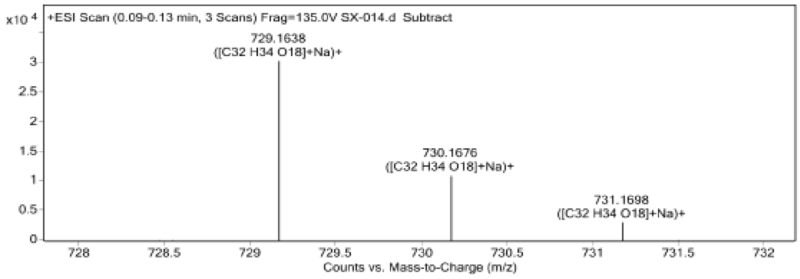

Peak List

m/z	z	Abund	Formula	Ion
202.1801		9926.41		
274.2742	1	26144.93		
318.3004	1	18852.47		
358.3685	1	13099.14		
729.1638	1	30382.15	C32 H34 O18	(M+Na)+
730.1676	1	11020.62	C32 H34 O18	(M+Na)+
736.5439	1	73769.88		
737.5471	1	38154.64		
1347.8837	1	16095.85		
1348.8862	1	15212.13		
1391.6109	1	21235.3		
1392.6146	1	17467.52		
1435.3375	1	12311.82		

Formula Calculator Element Limits

Element	Min	Max
C	3	60
H	0	120
O	0	30

Formula Calculator Results

Formula	CalculatedMass	CalculatedMz	Mz	Diff. (mDa)	Diff. (ppm)	DBE
C32 H34 O18	706.1745	729.1637	729.1638	-0.10	-0.14	16.0000

Figure S8. HRESIMS spectrum of compound **2**

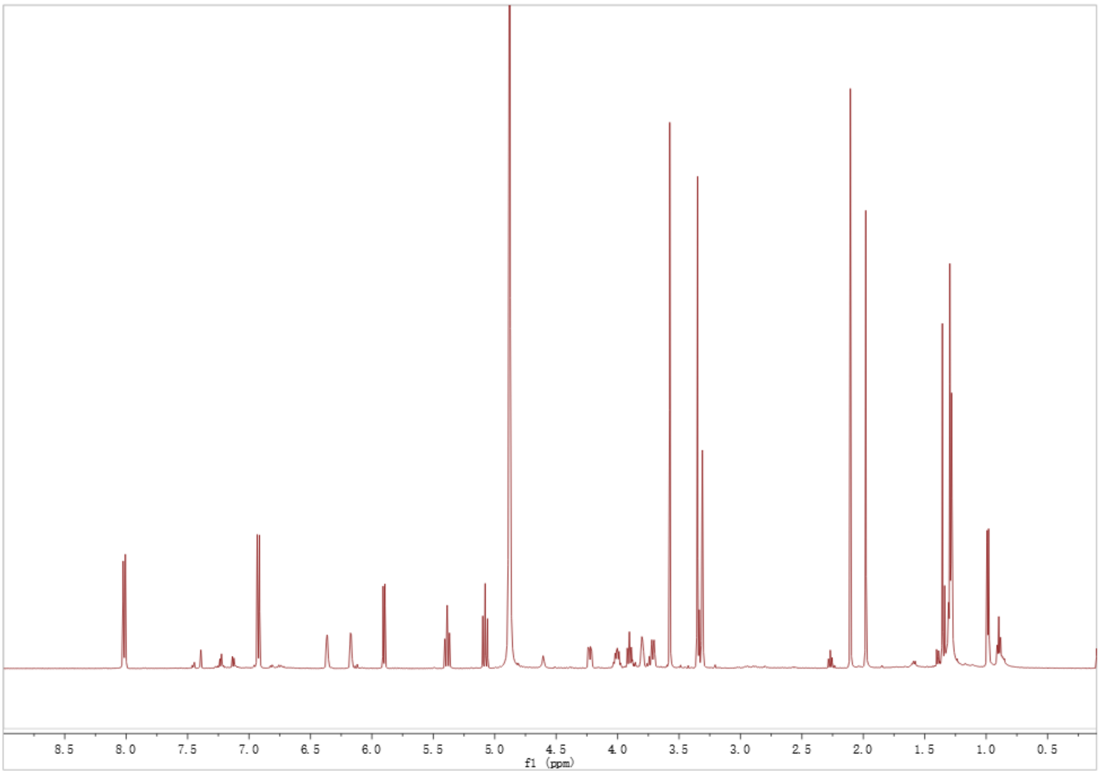

Figure S9. ^{1}H NMR spectrum of compound **2** in CD_3OD

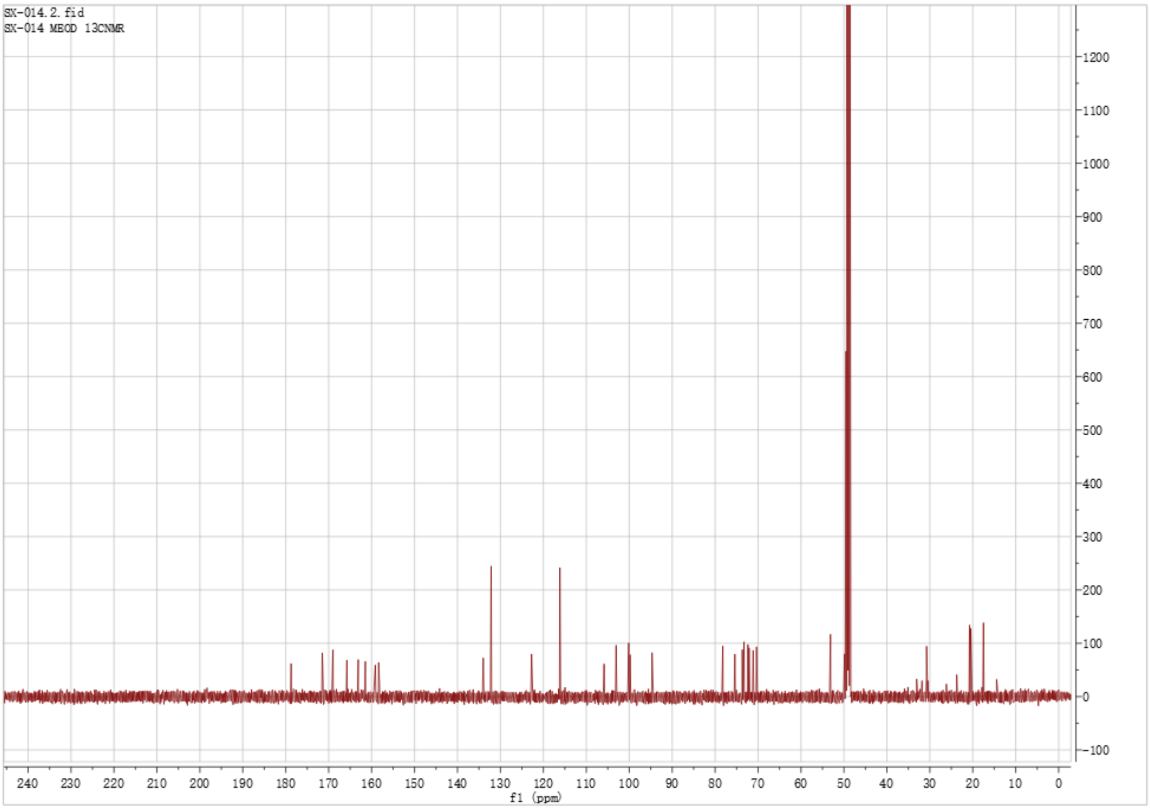

Figure S10. ^{13}C NMR spectrum of compound **2** in CD_3OD

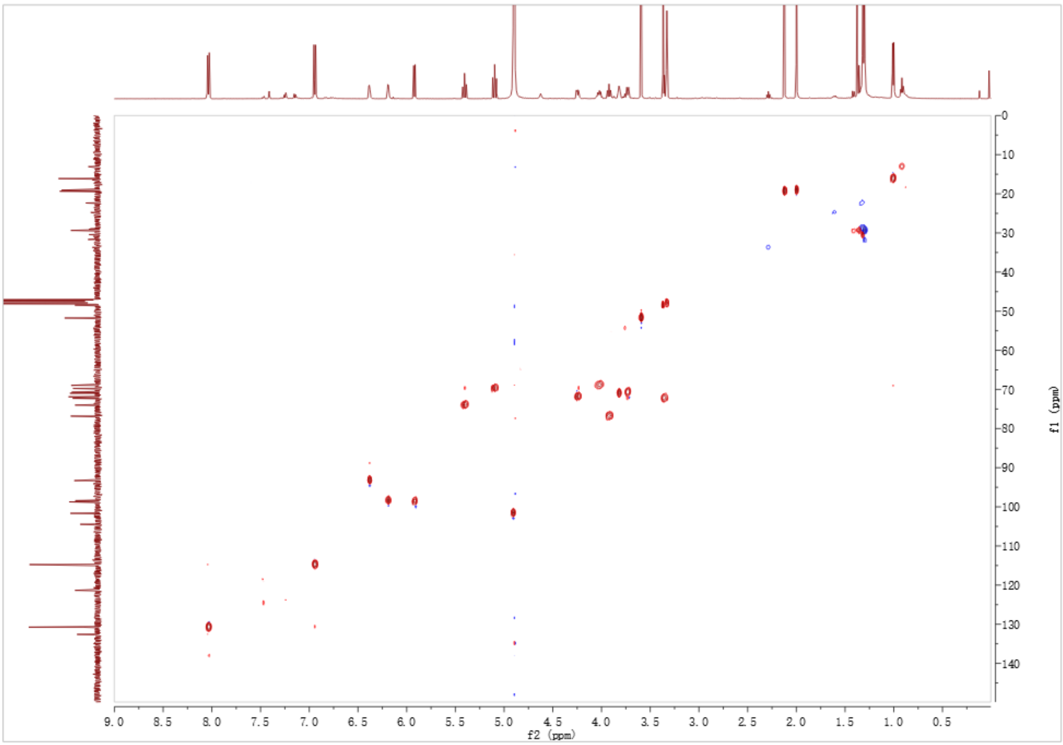

Figure S11. HSQC spectrum of compound **2** in CD_3OD

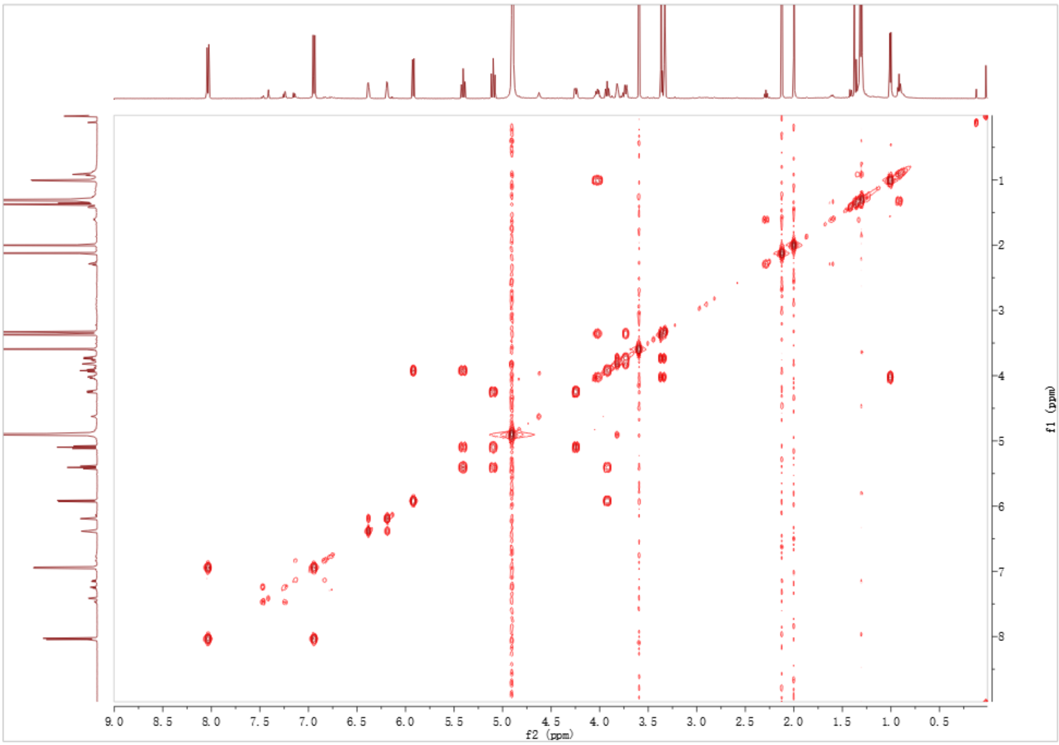

Figure S12. ^{1}H-^{1}H COSY spectrum of compound **2** in CD_3OD

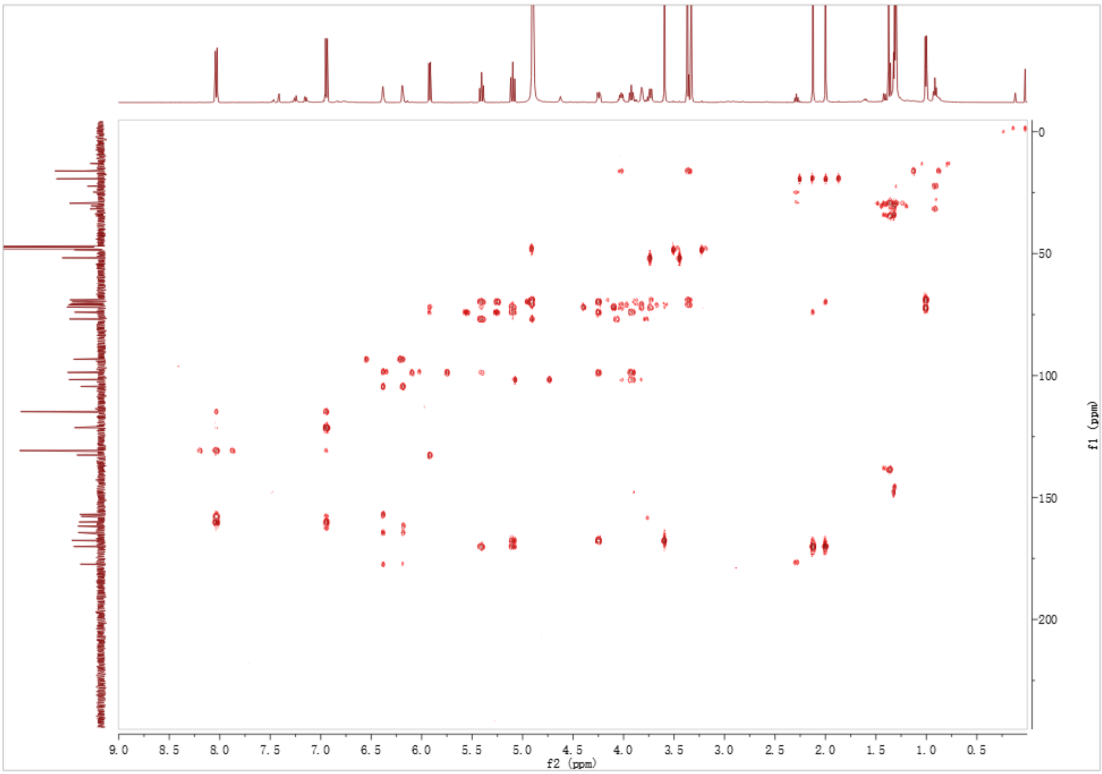

Figure S13. HMBC spectrum of compound **2** in CD_3OD

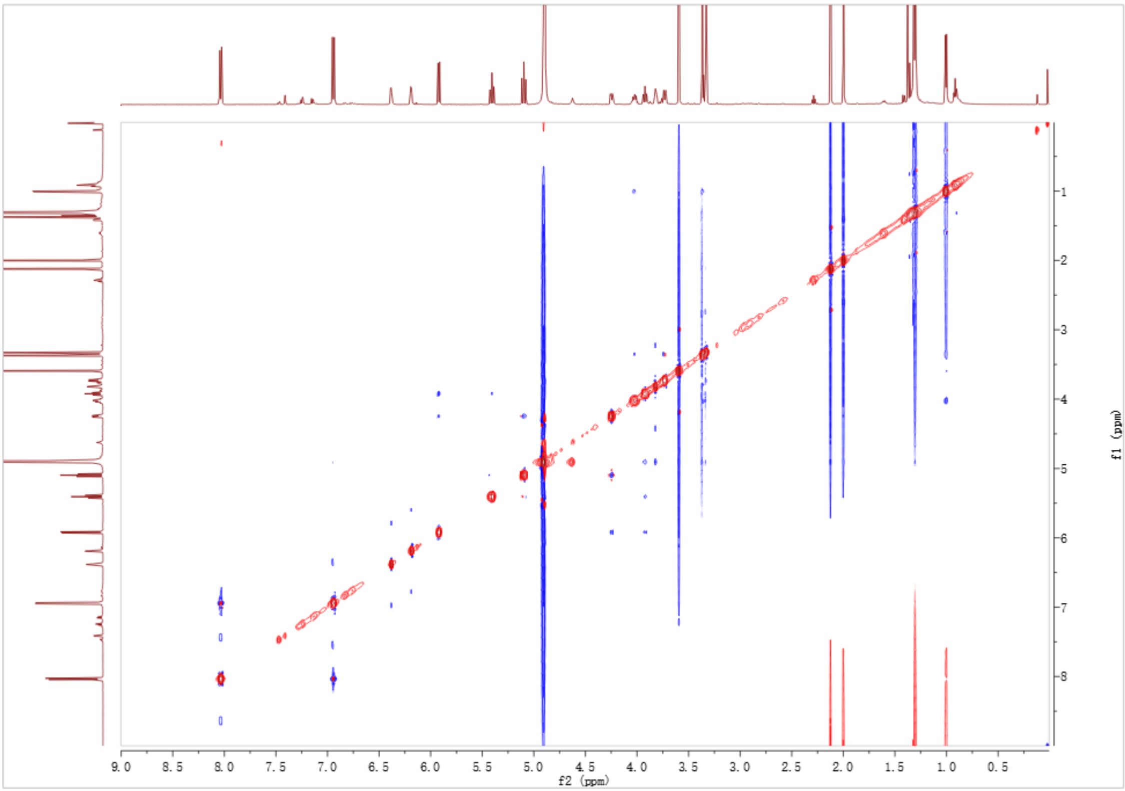

Figure S14. ROESY spectrum of compound **2** in CD_3OD

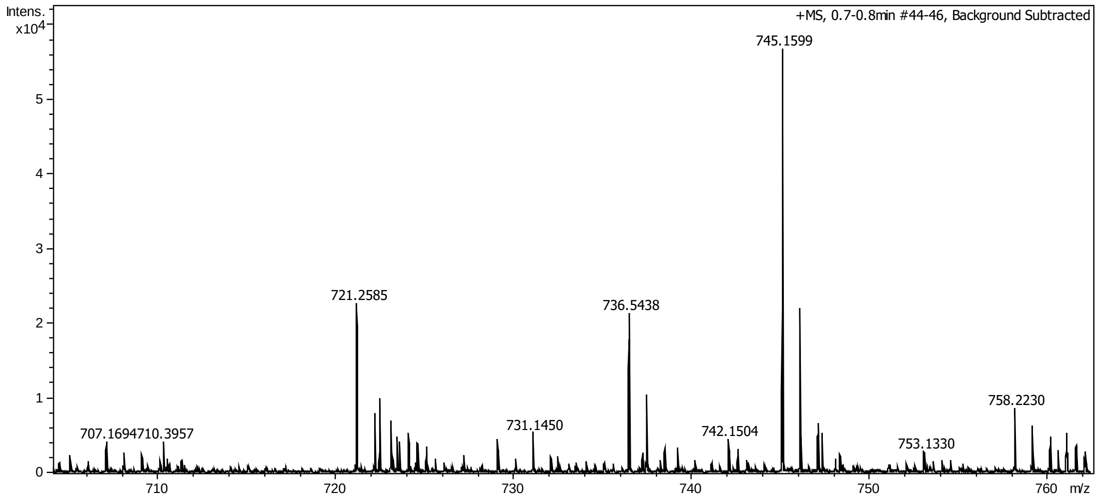

Meas. m/z	#	Ion Formula	m/z	err [ppm]	mSigma	# Sigma	Score	rdb	e⁻ Conf	N-Rule
745.1599	1	C34H33O19	745.1611	-1.5	7.6	1	100.00	18.5	even	ok
	2	C32H34NaO19	745.1586	-1.7	16.9	2	76.82	15.5	even	ok

Figure S15. HRESIMS spectrum of compound **3**

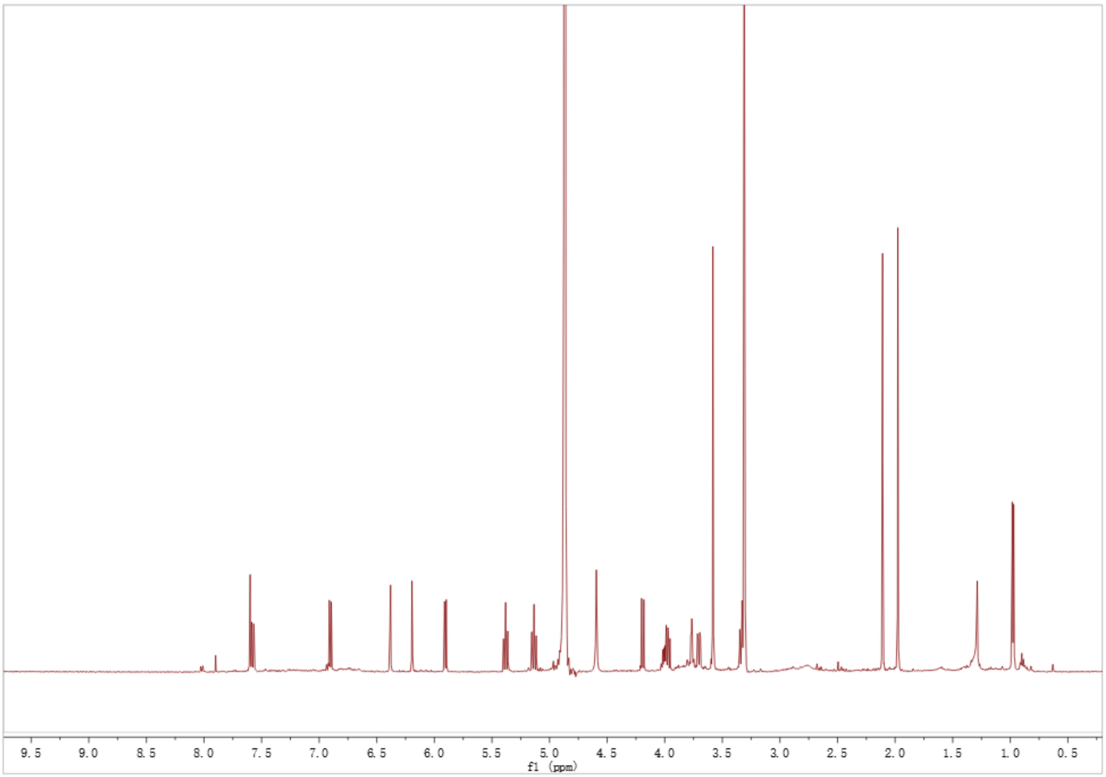

Figure S16. ^{1}H NMR spectrum of compound **3** in CD_3OD

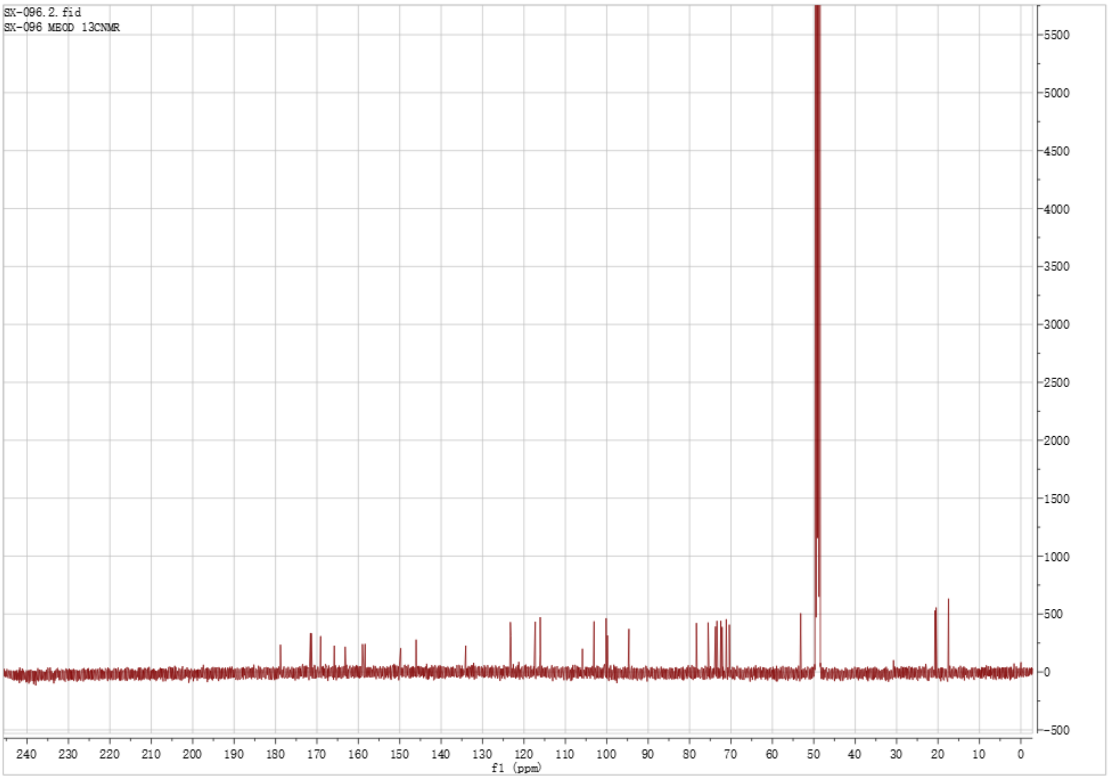

Figure S17. ^{13}C NMR spectrum of compound **3** in CD_3OD

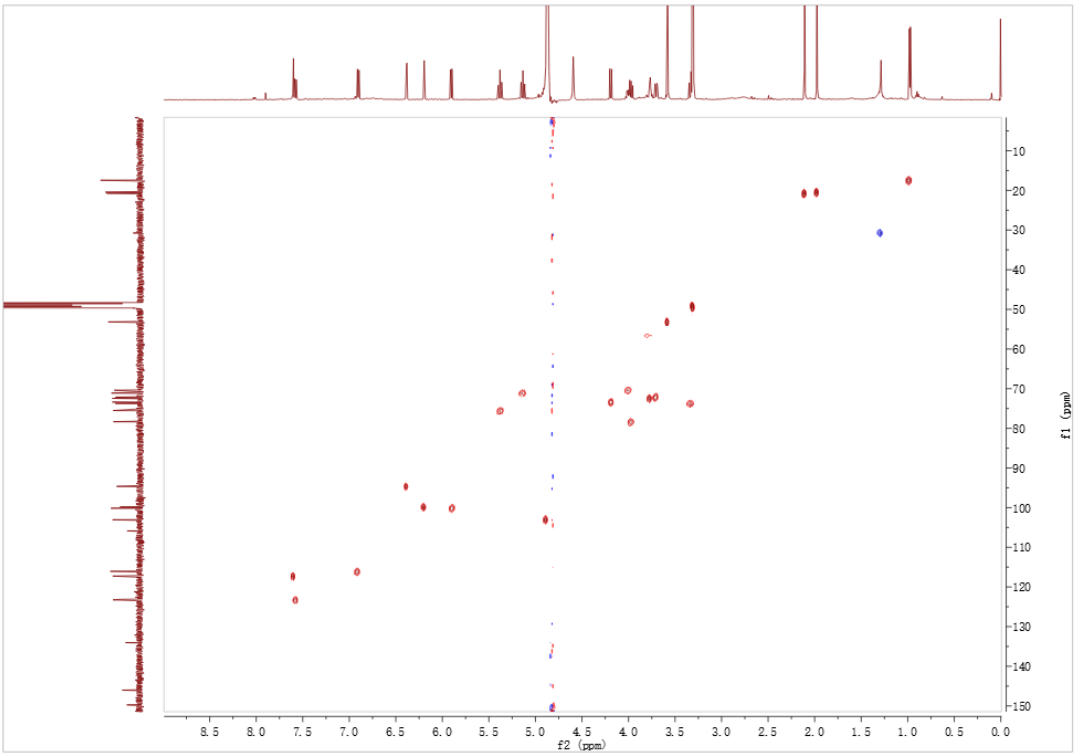

Figure S18. HSQC spectrum of compound **3** in CD_3OD

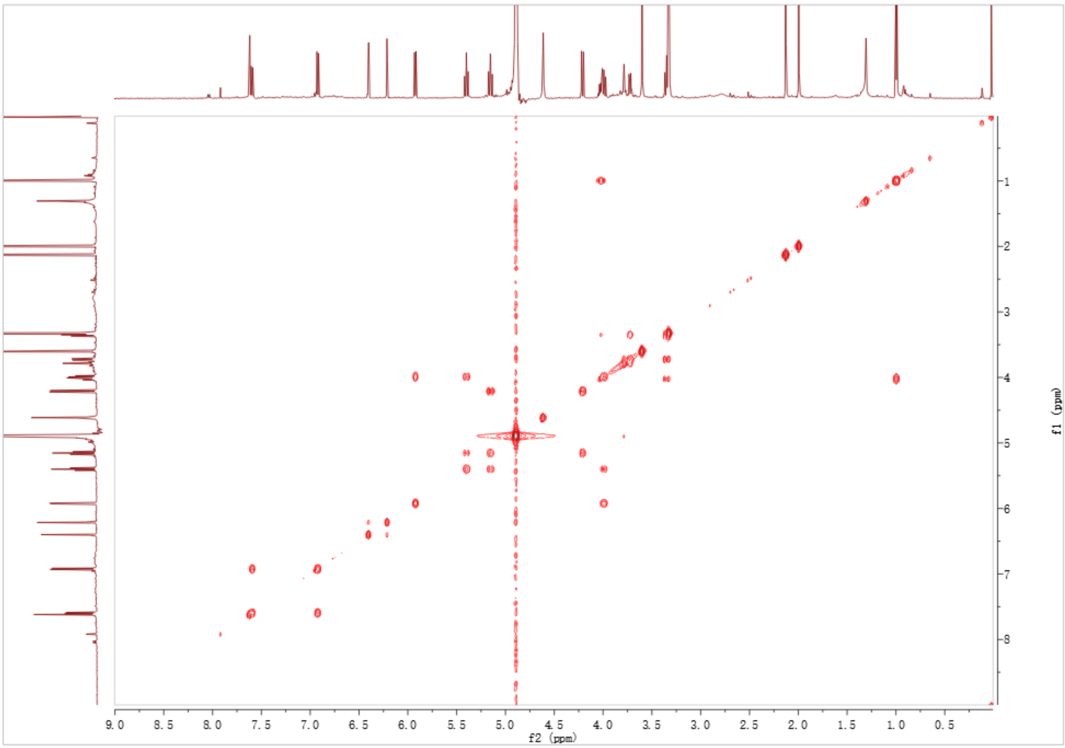

Figure S19. ^{1}H-^{1}H COSY spectrum of compound **3** in CD_3OD

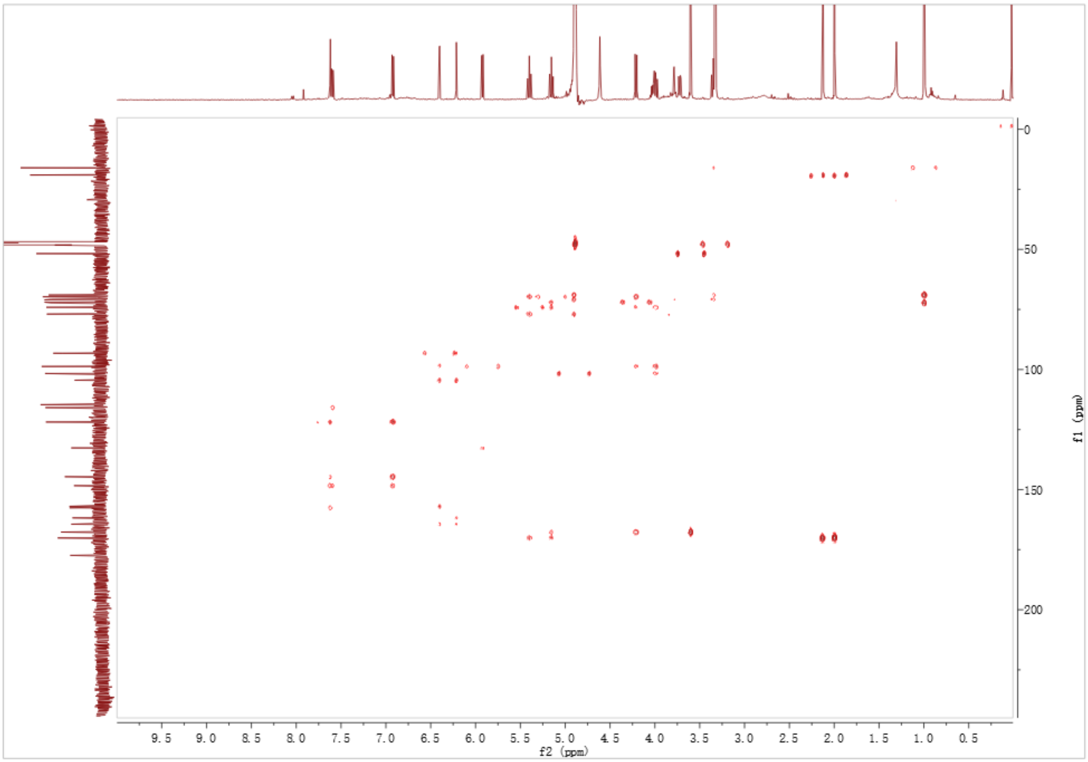

Figure S20. HMBC spectrum of compound **3** in CD_3OD

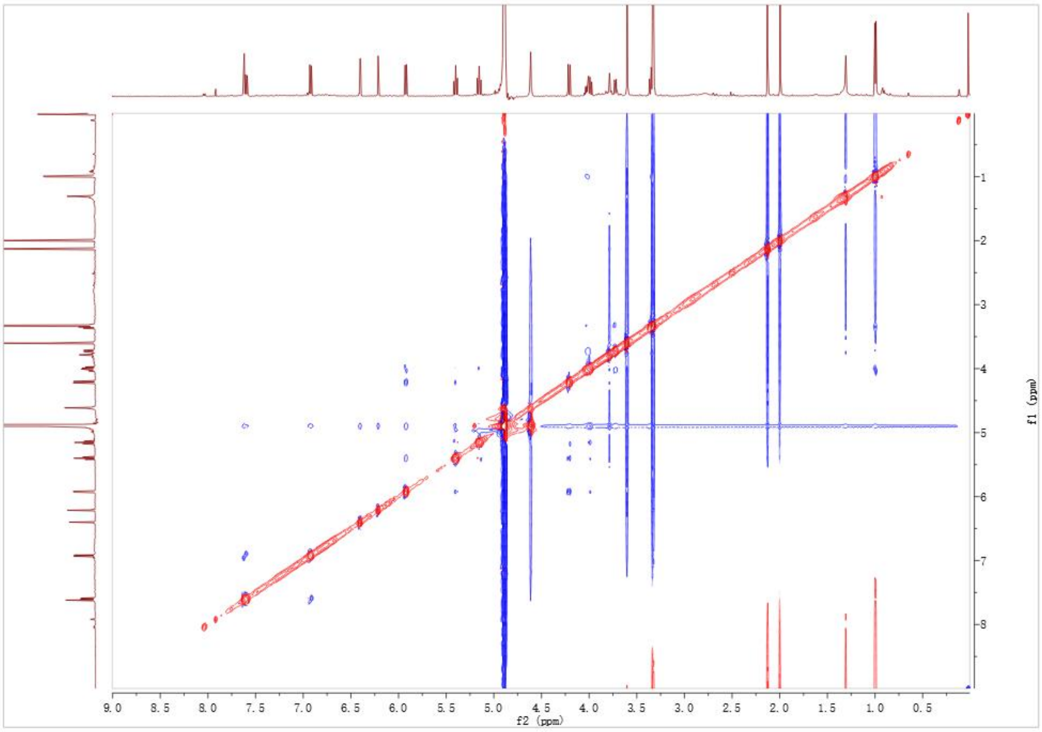

Figure S21. ROESY spectrum of compound **3** in CD_3OD

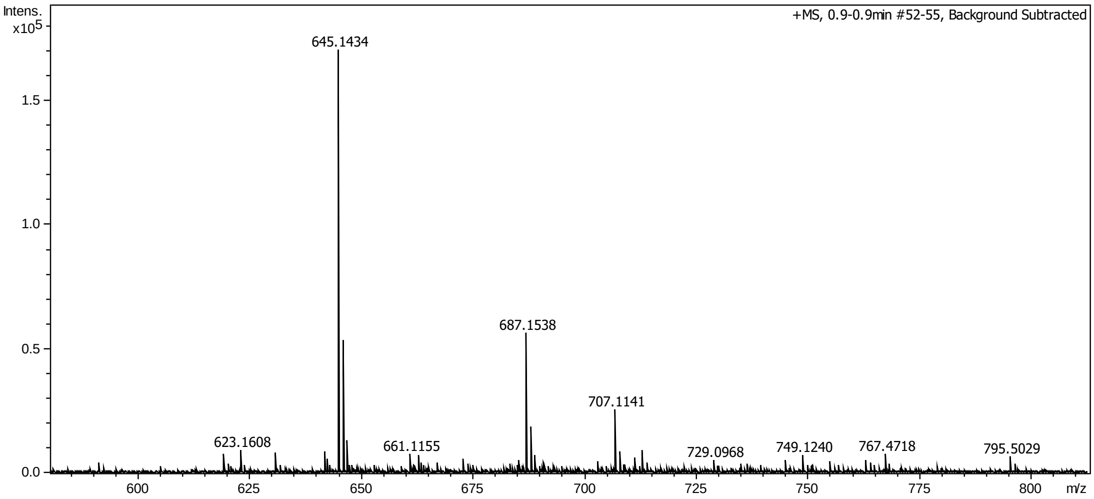

Meas. m/z	#	Ion Formula	m/z	err [ppm]	mSigma	# Sigma	Score	rdb	e⁻ Conf	N-Rule
623.1608	1	C28H31O16	623.1607	-0.2	14.9	1	100.00	13.5	even	ok
	2	C44H24NaO3	623.1618	1.5	84.5	2	7.96	32.5	even	ok
645.1434	1	C28H30NaO16	645.1426	-1.2	2.8	1	100.00	13.5	even	ok
687.1538	1	C30H32NaO17	687.1532	-0.9	21.4	1	100.00	14.5	even	ok
707.1141	1	C23H31O25	707.1149	-1.2	43.7	1	100.00	8.5	even	ok
	2	C39H24NaO12	707.1160	-2.7	49.0	2	52.66	27.5	even	ok

Figure S22. HRESIMS spectrum of compound **4**

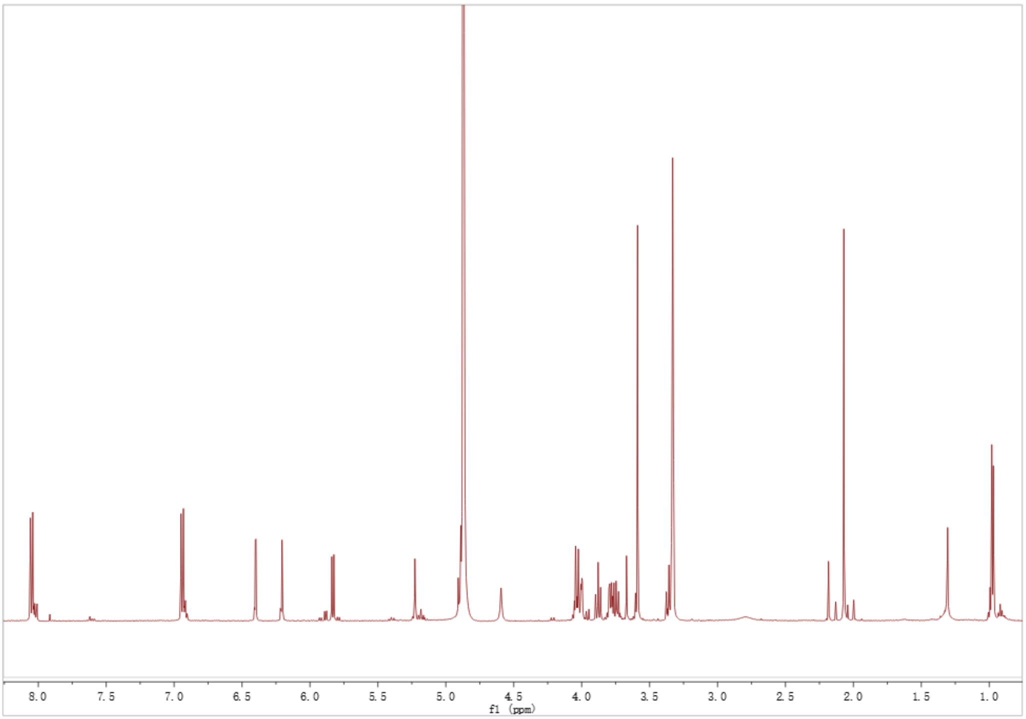

Figure S23. ^1H NMR spectrum of compound **4** in CD_3OD

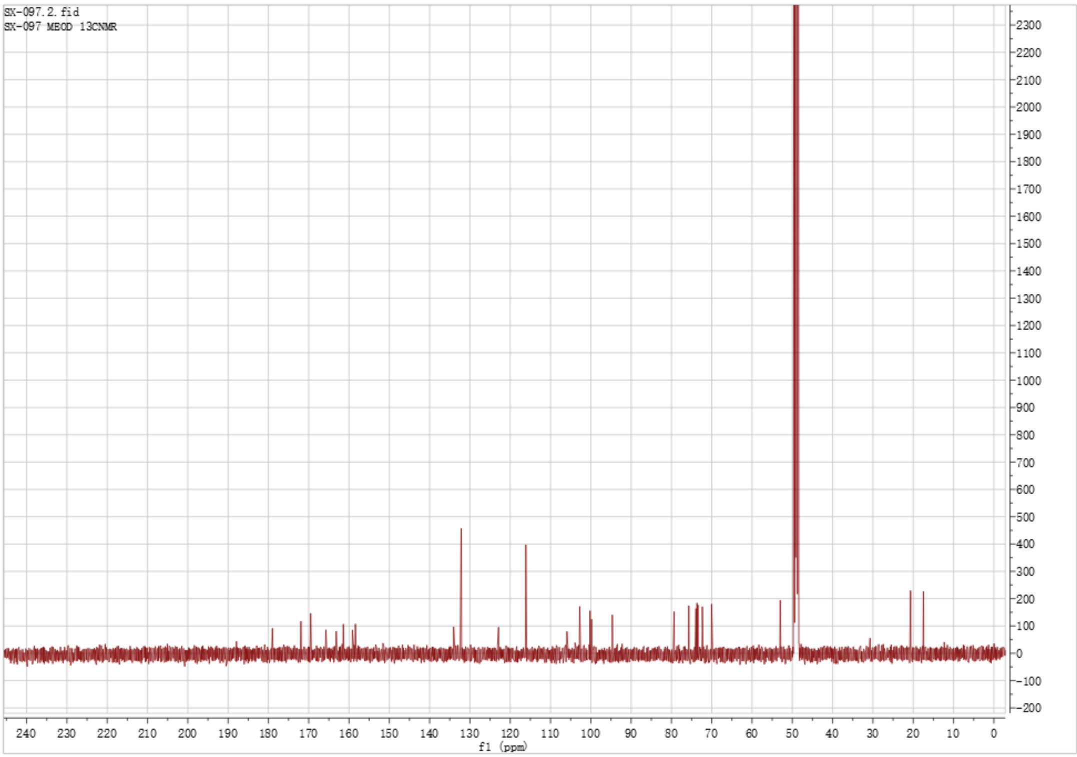

Figure S24. ^{13}C NMR spectrum of compound **4** in CD_3OD

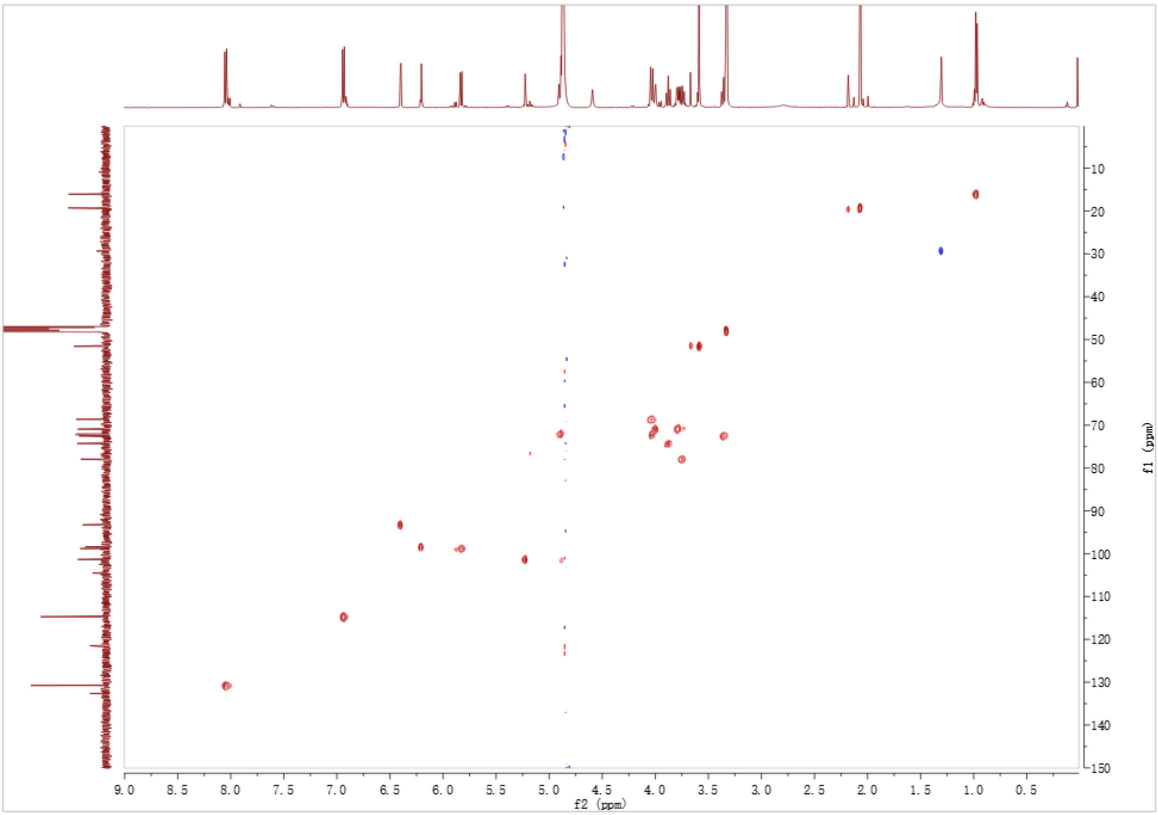

Figure S25. HSQC spectrum of compound **4** in CD_3OD

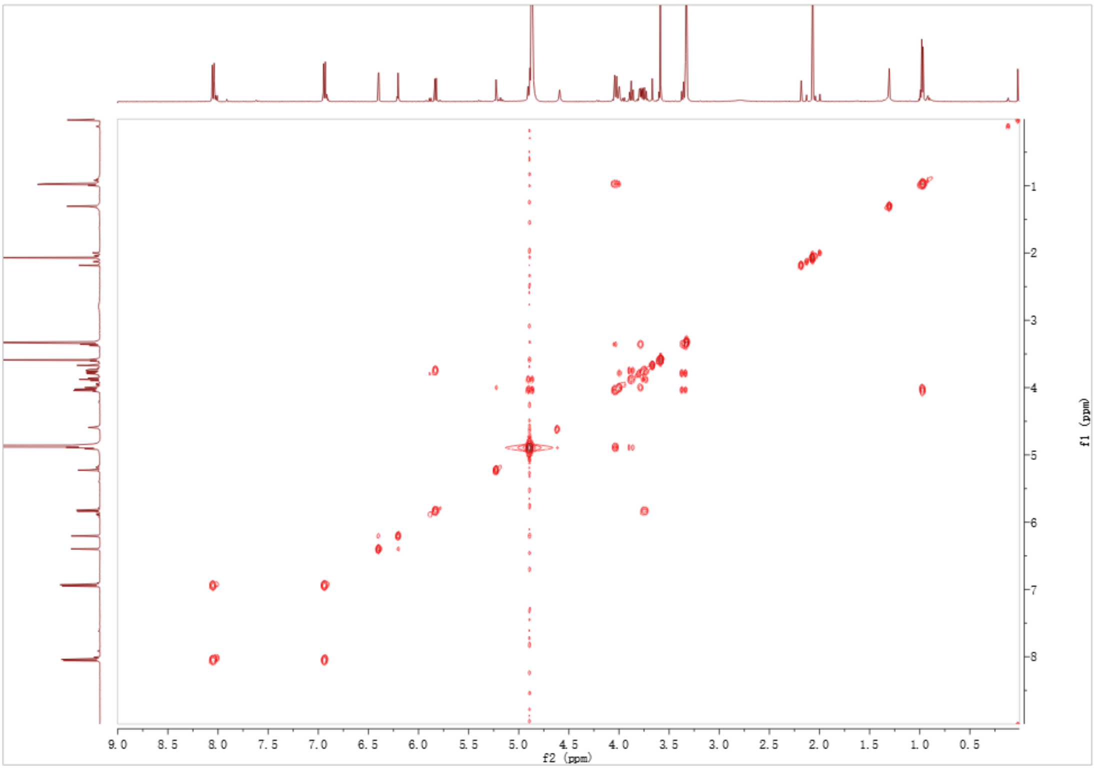

Figure S26. ^1H-^1H COSY spectrum of compound **4** in CD_3OD

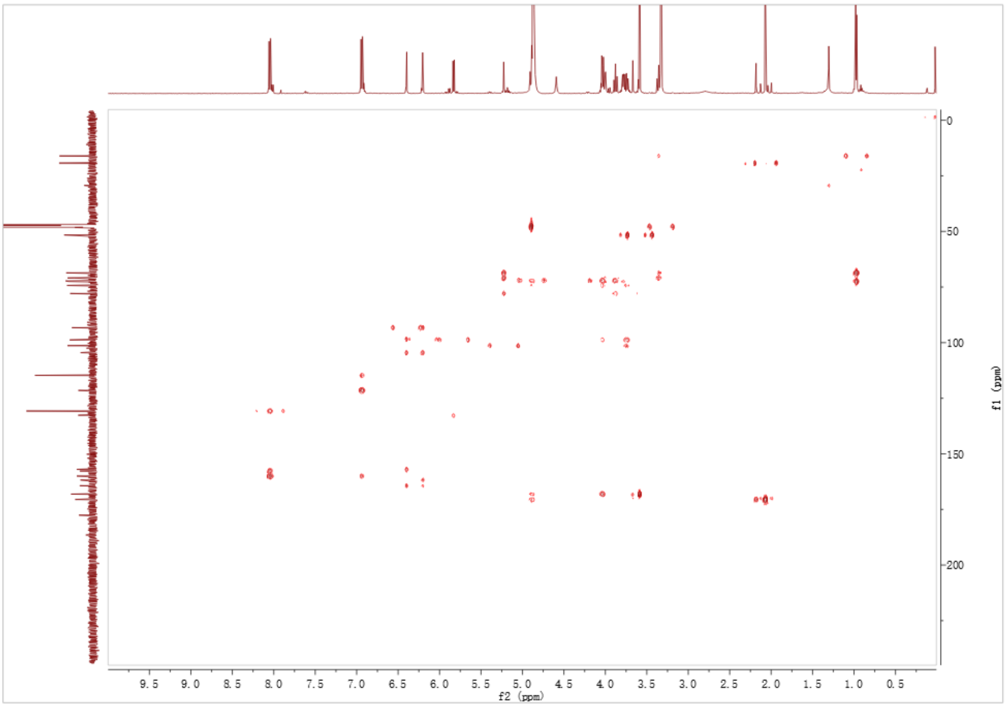

Figure S27. HMBC spectrum of compound **4** in CD_3OD

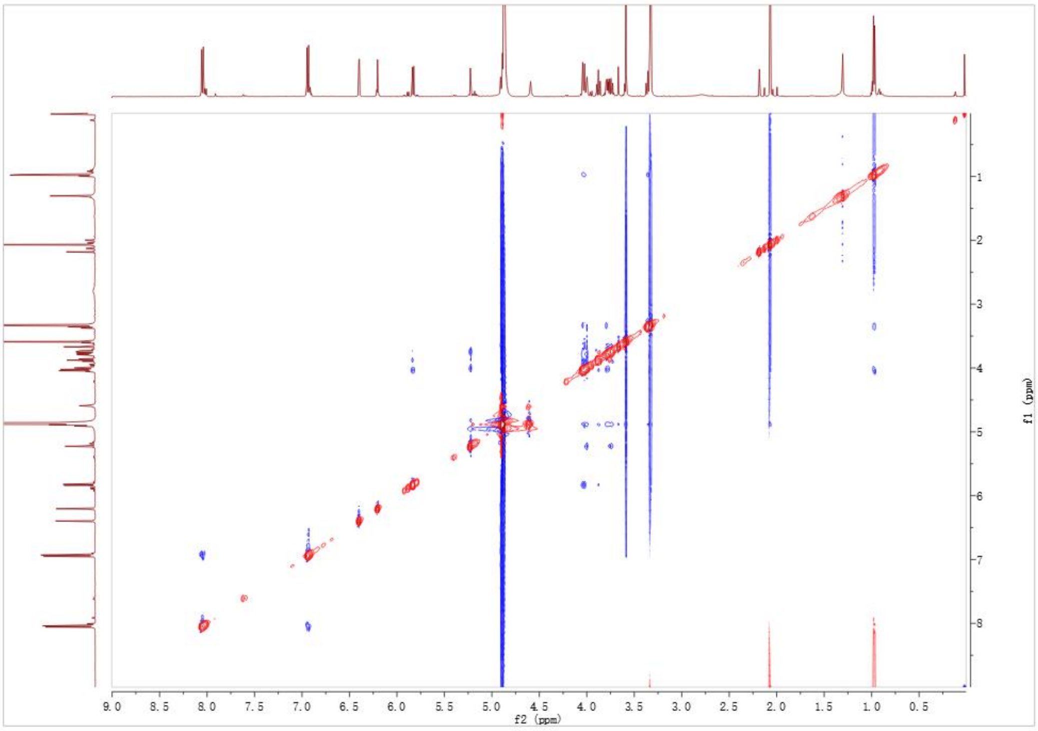

Figure S28. ROESY spectrum of compound **4** in CD_3OD

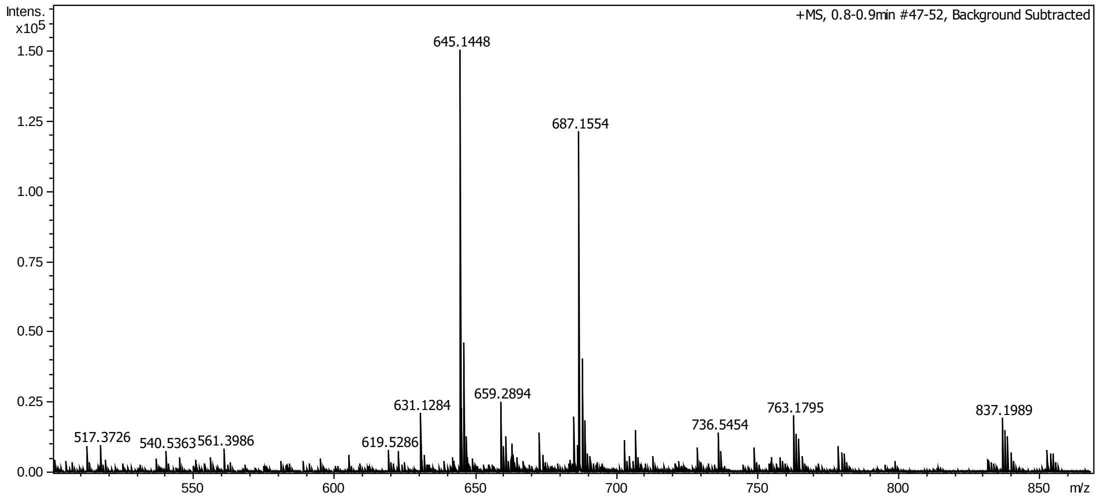

Figure S29. HRESIMS spectrum of compound **5**

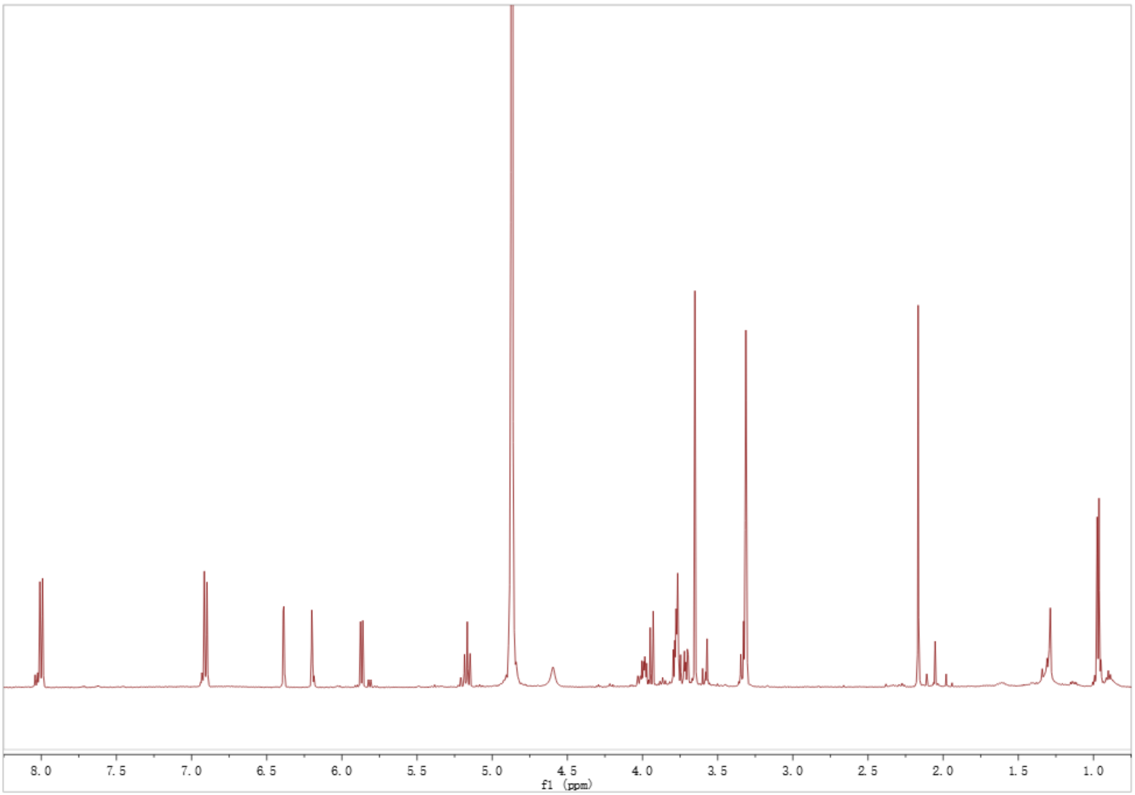

Figure S30. ^{1}H NMR spectrum of compound **5** in CD_3OD

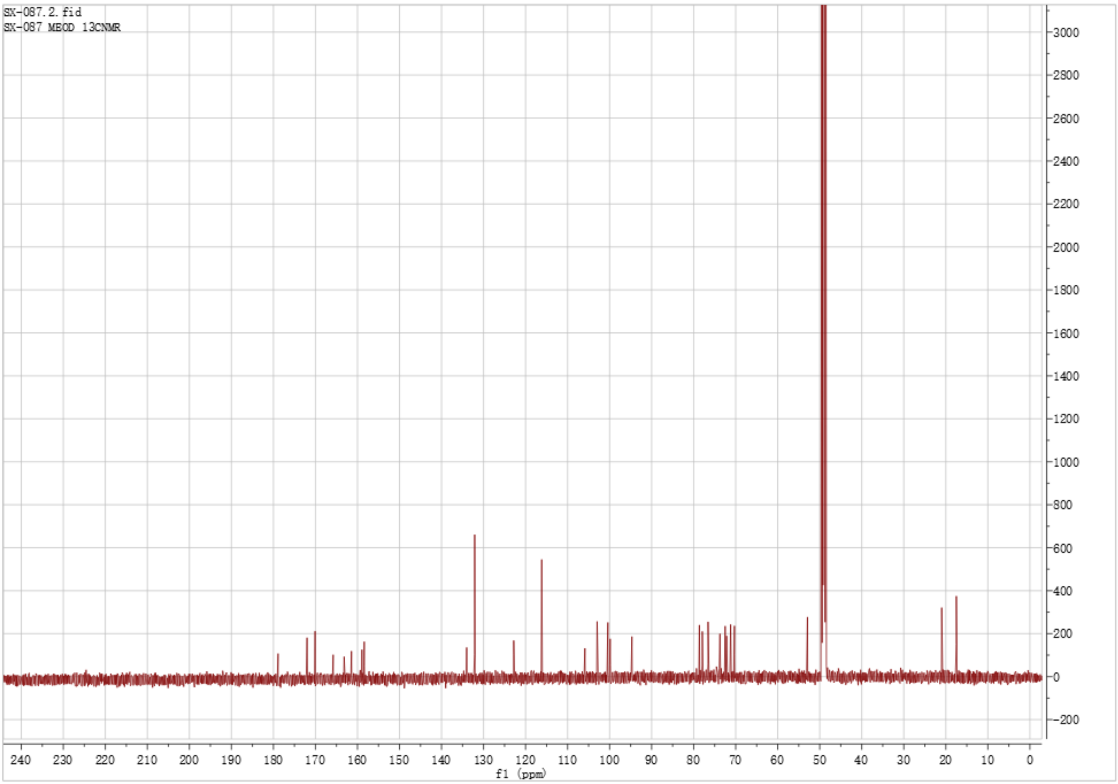

Figure S31. ^{13}C NMR spectrum of compound **5** in CD_3OD

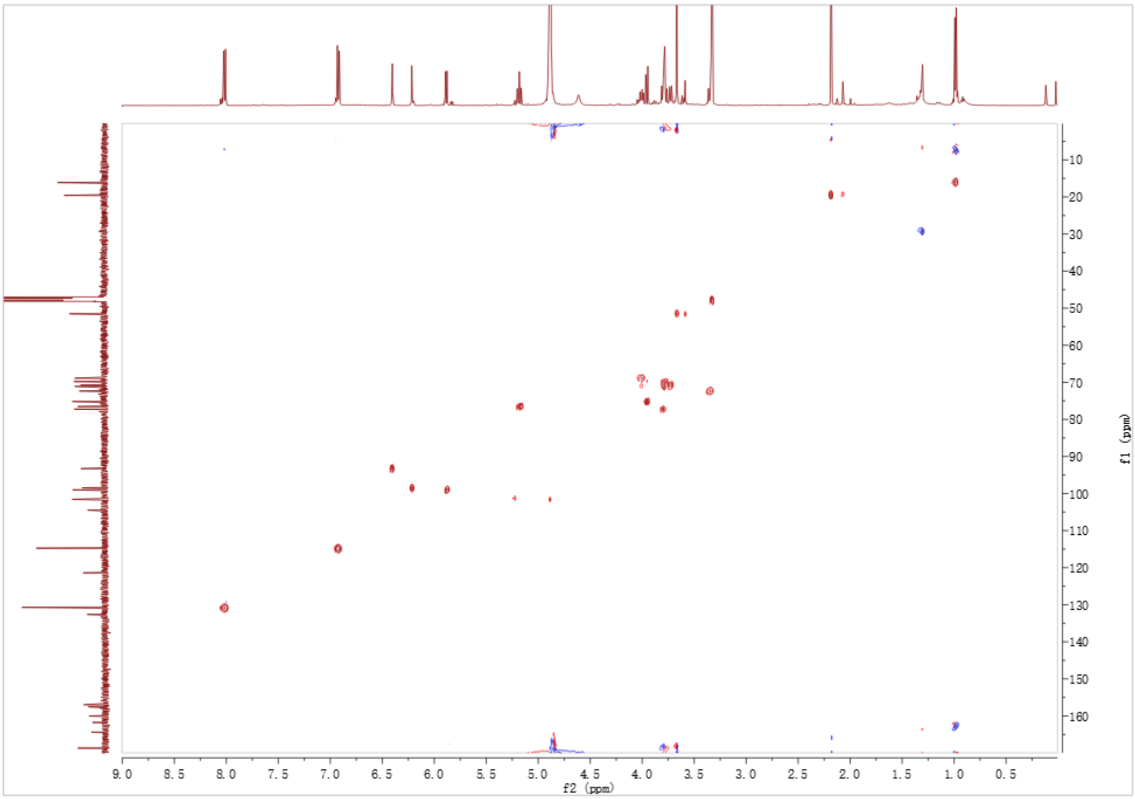

Figure S32. HSQC spectrum of compound **5** in CD_3OD

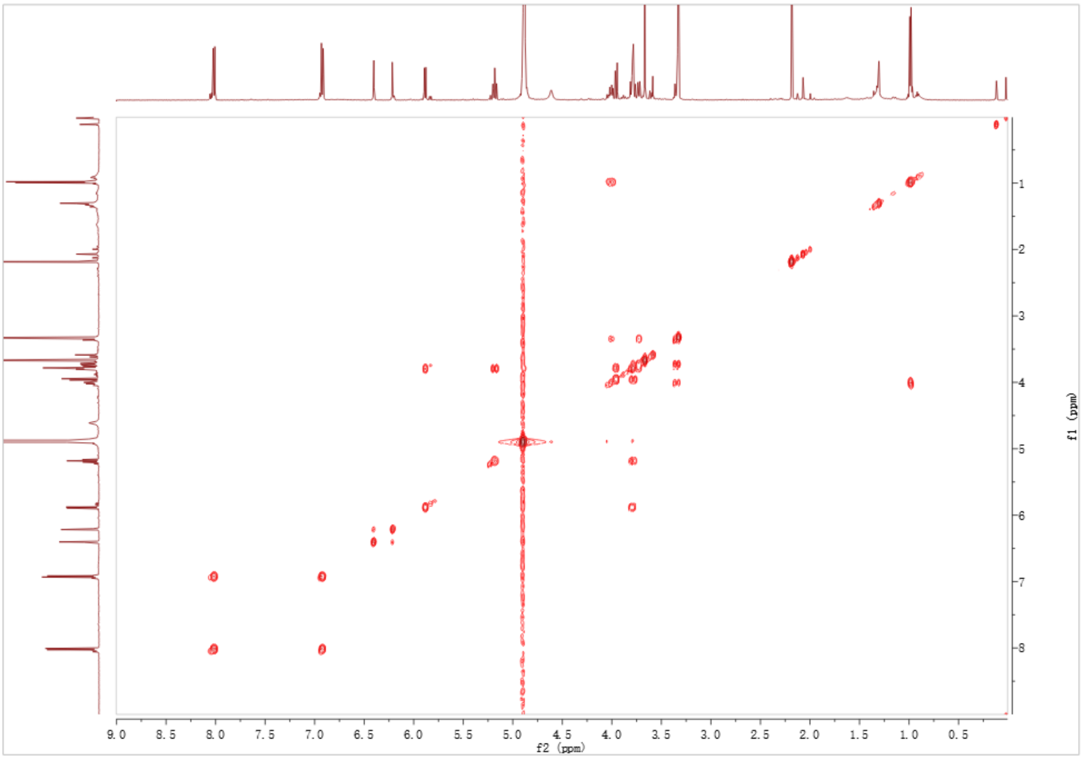

Figure S33. ^{1}H-^{1}H COSY spectrum of compound **5** in CD_3OD

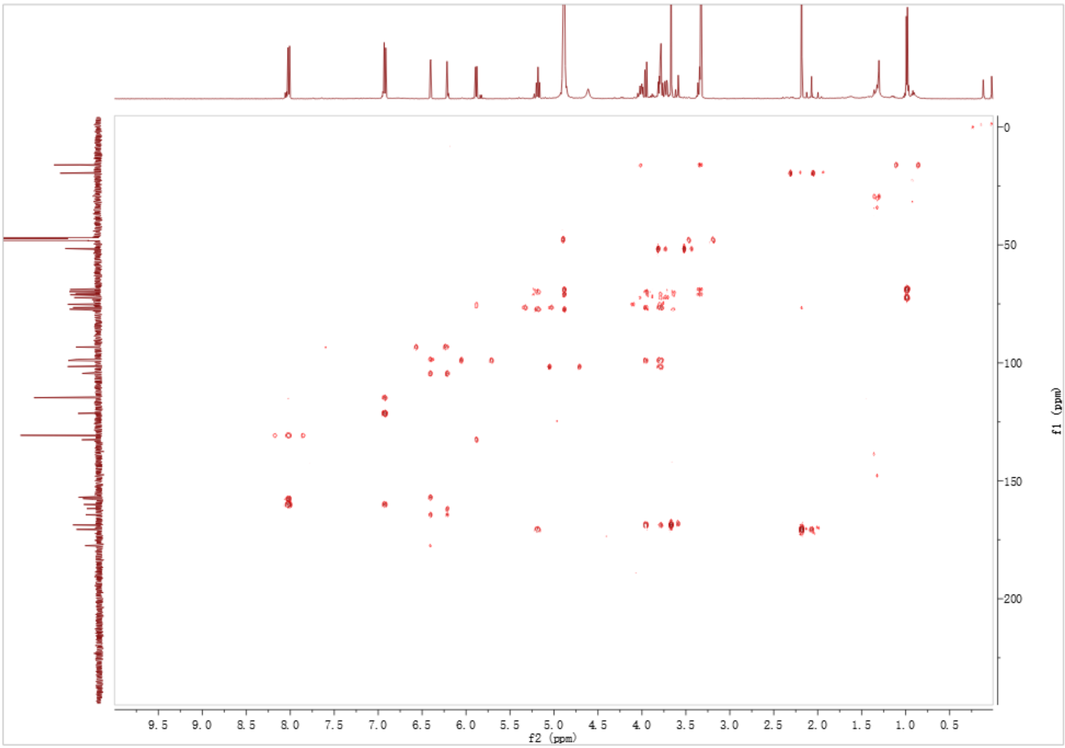

Figure S34. HMBC spectrum of compound **5** in CD_3OD

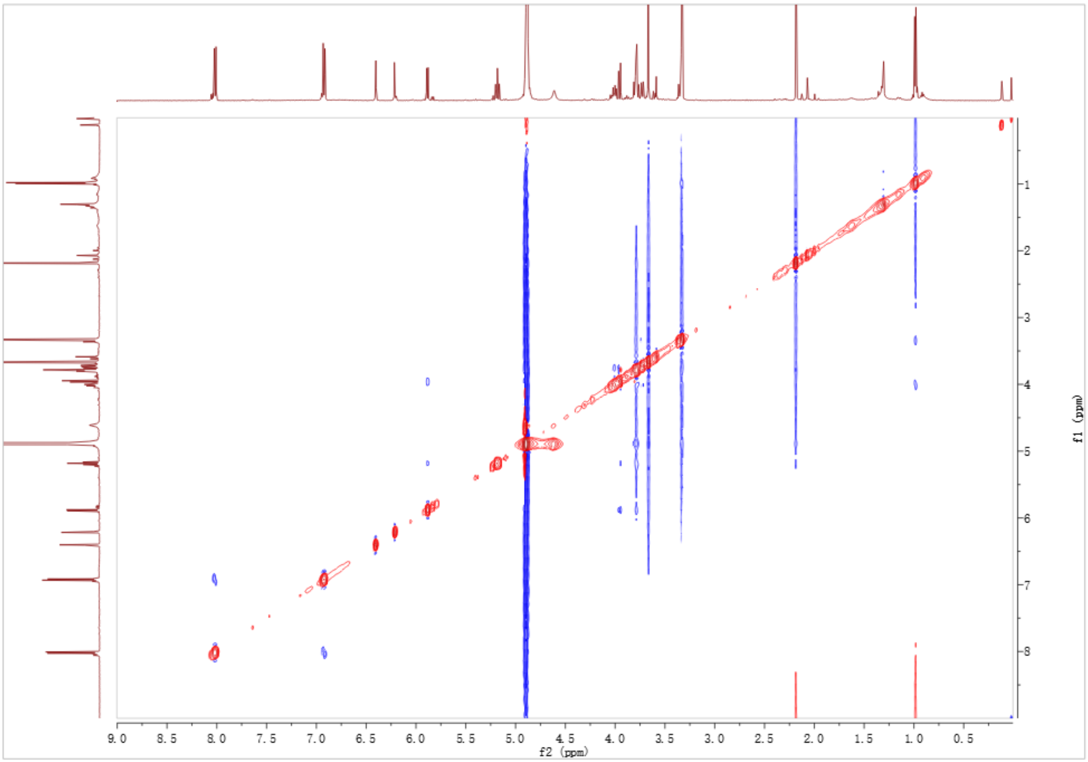

Figure S35. ROESY spectrum of compound **5** in CD_3OD

Computational details

Compound 2

symmetry c1

C	5.686693000	3.457778000	-1.747269000
C	4.311634000	3.296187000	-1.598661000
C	3.788161000	2.259580000	-0.771249000
C	4.731331000	1.432207000	-0.120261000
C	6.108338000	1.580388000	-0.257928000
C	6.582273000	2.603647000	-1.082518000
H	6.069332000	4.257134000	-2.387380000
H	6.792317000	0.913157000	0.265210000
C	3.022835000	0.139616000	0.929910000
C	2.056395000	0.897314000	0.329380000
O	7.921527000	2.737185000	-1.213012000
H	8.129407000	3.467411000	-1.818719000
O	3.447178000	4.115840000	-2.234350000
H	3.945805000	4.769562000	-2.751137000
O	4.331453000	0.420206000	0.701153000
C	2.360205000	2.005945000	-0.575909000
O	1.440983000	2.629240000	-1.125431000
C	2.842299000	-1.023831000	1.804966000
C	1.716891000	-1.168897000	2.640314000
C	3.802683000	-2.053196000	1.789225000
C	1.547139000	-2.308533000	3.414516000
H	0.966677000	-0.381547000	2.678746000
C	3.633427000	-3.202055000	2.555118000
H	4.682947000	-1.961539000	1.151887000
C	2.498556000	-3.340204000	3.367696000
H	0.675610000	-2.420540000	4.062194000
H	4.373109000	-4.006172000	2.523630000
O	2.276960000	-4.444179000	4.124515000
H	3.000709000	-5.078445000	3.997506000
O	0.719237000	0.637525000	0.539702000
C	0.097673000	-0.149955000	-0.439474000
C	-1.410350000	0.014699000	-0.283403000
H	0.409213000	0.158043000	-1.453648000
C	-0.104744000	-2.433608000	-1.050360000
C	-2.152871000	-1.018673000	-1.147437000
H	-1.661463000	-0.152626000	0.775049000
C	-1.638355000	-2.424161000	-0.820627000
H	0.103084000	-2.209424000	-2.111075000
H	-1.953220000	-0.787818000	-2.203226000
H	-1.807887000	-2.653636000	0.240290000
O	0.491644000	-1.485855000	-0.199464000
C	0.499787000	-3.784033000	-0.703723000
O	0.954316000	-4.073521000	0.379928000
O	0.423238000	-4.612106000	-1.742249000
C	0.784133000	-5.983593000	-1.508518000
H	0.143730000	-6.413170000	-0.725154000
H	0.624890000	-6.503404000	-2.459558000
H	1.838122000	-6.053907000	-1.205444000
C	-2.332597000	-3.482641000	-1.650828000
O	-2.766103000	-3.303880000	-2.770374000
O	-2.388543000	-4.647576000	-1.006511000
C	-3.647413000	-0.916177000	-0.913893000
O	-4.239156000	-1.498283000	-0.026310000
O	-4.234482000	-0.110159000	-1.794655000
C	-2.892523000	-5.773963000	-1.741519000
H	-2.833735000	-6.629618000	-1.059784000
H	-3.934274000	-5.597297000	-2.044325000
H	-2.274124000	-5.951937000	-2.632750000
C	-5.643697000	0.113181000	-1.633102000
H	-5.840612000	0.604329000	-0.670535000
H	-5.941322000	0.767232000	-2.460471000
H	-6.191169000	-0.838604000	-1.682519000
O	-1.772975000	1.324888000	-0.690249000
C	-2.278752000	2.135213000	0.324435000
C	-2.461599000	3.550172000	-0.206309000
H	-1.593795000	2.144430000	1.194718000
C	-3.158467000	4.394110000	0.854460000
H	-3.116867000	3.485235000	-1.096086000
C	-4.143906000	2.296424000	1.803063000
C	-4.438353000	3.734112000	1.351221000
H	-2.470454000	4.484174000	1.718213000
H	-3.430198000	2.341579000	2.647974000
H	-5.160631000	3.696483000	0.515449000
O	-1.198396000	4.091079000	-0.537296000
H	-1.338289000	5.032982000	-0.721966000
O	-3.404967000	5.669809000	0.288871000
H	-3.836291000	6.201986000	0.975298000
O	-4.936525000	4.537127000	2.409790000
H	-5.881135000	4.352499000	2.512754000
O	-3.530889000	1.603685000	0.712062000
C	-5.380857000	1.525842000	2.212309000
H	-5.837014000	1.990824000	3.098596000
H	-6.123897000	1.521833000	1.399399000
H	-5.120408000	0.485228000	2.456157000

Compound 6

C	4.995563000	2.145064000	-0.027261000
C	3.785055000	1.433714000	-0.014226000
C	3.772997000	0.036151000	-0.023414000
C	5.008475000	-0.645594000	-0.046541000
C	6.211754000	0.054319000	-0.059289000
C	6.208890000	1.469108000	-0.049621000
H	5.004094000	3.237309000	-0.019870000
H	2.851600000	1.996863000	0.003113000
H	5.012240000	-1.736357000	-0.052927000
C	2.502308000	-0.788443000	-0.009307000
H	2.489867000	-1.441307000	-0.899245000
H	2.533800000	-1.479377000	0.849445000
C	1.203078000	0.000784000	0.042945000
H	1.098914000	0.681236000	-0.818811000
H	1.171693000	0.657912000	0.931129000
C	-1.357587000	-0.139463000	-0.061319000
H	-1.382203000	0.215943000	-1.108715000
C	-2.588953000	-0.979353000	0.252529000
H	-2.535966000	-1.329320000	1.298031000
H	-2.579319000	-1.888318000	-0.371946000
C	-3.893863000	-0.217955000	0.032287000
H	-3.902480000	0.694303000	0.654557000
H	-3.950705000	0.125933000	-1.015608000
C	-5.140046000	-1.055534000	0.353798000
H	-5.086046000	-1.387761000	1.403858000
H	-5.132184000	-1.963574000	-0.271496000
C	-6.423295000	-0.295139000	0.127780000
C	-7.068975000	-0.326397000	-1.118728000
C	-6.971074000	0.507799000	1.141466000
C	-8.229639000	0.419192000	-1.346392000
H	-6.655836000	-0.947261000	-1.918749000
C	-8.131104000	1.255474000	0.918739000
H	-6.481011000	0.542832000	2.118630000
C	-8.765049000	1.214070000	-0.327611000
H	-8.719572000	0.377497000	-2.322586000
H	-8.543626000	1.871010000	1.722290000
H	-9.673402000	1.795634000	-0.503005000
O	7.391297000	2.154746000	-0.062039000
H	8.111268000	1.500890000	-0.077161000
O	7.458232000	-0.503390000	-0.080468000
C	7.572126000	-1.918179000	-0.086081000
H	8.645946000	-2.144462000	-0.101043000
H	7.119014000	-2.359499000	0.817357000
H	7.095312000	-2.354435000	-0.979744000
C	-0.039479000	-0.858321000	0.111614000
O	0.016862000	-2.065617000	0.298266000
H	-1.329611000	0.780622000	0.548217000

Compound 7

C	6.381671000	0.370612000	-1.741344000
C	5.492785000	-0.704099000	-1.594937000
C	4.780738000	-0.890503000	-0.406616000
C	4.967600000	0.026543000	0.646839000
C	5.848151000	1.097627000	0.507000000
C	6.563778000	1.274439000	-0.699785000
H	6.942762000	0.517530000	-2.666904000
H	5.357789000	-1.405877000	-2.421666000
H	4.416719000	-0.110602000	1.578216000
C	3.783090000	-2.013306000	-0.254986000
H	3.998028000	-2.805889000	-0.987947000
H	3.875908000	-2.469860000	0.742846000
C	2.342521000	-1.532675000	-0.439815000
H	2.195561000	-1.078333000	-1.434623000
H	2.109710000	-0.732369000	0.283924000
C	-0.134464000	-2.268504000	-0.598944000
H	-0.775084000	-3.077357000	-0.215616000
C	-0.613458000	-0.917084000	-0.078036000
H	-0.021944000	-0.114065000	-0.555317000
C	-2.089095000	-0.682398000	-0.397667000
H	-2.230225000	-0.759383000	-1.488484000
H	-2.682676000	-1.491146000	0.062026000
C	-2.609897000	0.678043000	0.085950000
H	-2.008693000	1.478519000	-0.375316000
H	-2.462118000	0.749200000	1.176319000
C	-4.069498000	0.890583000	-0.235037000
C	-5.059371000	0.308537000	0.581288000
C	-4.468642000	1.613047000	-1.363630000
C	-6.410641000	0.451677000	0.270497000
H	-4.758592000	-0.256337000	1.464410000
C	-5.826180000	1.761909000	-1.681108000
H	-3.713414000	2.070481000	-2.007591000
C	-6.801152000	1.185339000	-0.873223000
H	-6.141793000	2.329004000	-2.559692000
O	7.427587000	2.325644000	-0.830762000
H	7.404269000	2.828783000	0.001351000
O	6.111045000	2.041796000	1.458303000

C	5.431104000	1.962295000	2.701630000
H	5.780491000	2.811289000	3.303050000
H	4.339204000	2.036518000	2.565571000
H	5.667097000	1.022042000	3.227407000
C	1.299615000	-2.606956000	-0.245747000
O	1.594448000	-3.720394000	0.163581000
O	-8.127491000	1.325798000	-1.172854000
H	-8.633643000	0.847599000	-0.493802000
O	-7.451218000	-0.063350000	0.990216000
C	-7.162620000	-0.833483000	2.146953000
H	-6.622283000	-0.236718000	2.900671000
H	-8.128962000	-1.149997000	2.559952000
H	-6.564270000	-1.724934000	1.894811000
H	-0.209135000	-2.271258000	-1.700140000
O	-0.370121000	-0.913130000	1.331199000
H	-0.484909000	-0.007616000	1.653396000

Compound 8

C	6.413160000	1.336676000	-1.630129000
C	5.346914000	0.446846000	-1.822669000
C	4.914708000	-0.390897000	-0.790034000
C	5.570434000	-0.325758000	0.455128000
C	6.630986000	0.556969000	0.652844000
C	7.057989000	1.399608000	-0.398931000
H	6.756040000	1.990171000	-2.435403000
H	4.848667000	0.407849000	-2.794517000
H	5.241181000	-0.975592000	1.266870000
C	3.732616000	-1.312327000	-0.971169000
H	3.606542000	-1.554283000	-2.037569000
H	3.912909000	-2.265694000	-0.449687000
C	2.435243000	-0.700682000	-0.442784000
H	2.209163000	0.252507000	-0.954110000
H	2.529707000	-0.431673000	0.625038000
C	-0.052496000	-1.021644000	-0.076851000
H	-0.021068000	-0.008145000	0.334973000
C	-1.206047000	-1.710874000	-0.113183000
H	-1.184456000	-2.721329000	-0.540667000
C	-2.527854000	-1.210344000	0.361711000
H	-2.891746000	-1.875233000	1.165986000
H	-2.427096000	-0.201297000	0.791509000
C	-3.589015000	-1.197519000	-0.760840000
H	-3.685133000	-2.215215000	-1.172156000
H	-3.232418000	-0.549715000	-1.577111000
C	-4.931636000	-0.713719000	-0.270989000
C	-5.248769000	0.653825000	-0.291039000
C	-5.867772000	-1.605566000	0.274805000
C	-6.464769000	1.107362000	0.214821000
H	-4.543030000	1.380747000	-0.700744000
C	-7.089829000	-1.158039000	0.784595000
H	-5.637358000	-2.673842000	0.301880000
C	-7.399858000	0.203901000	0.751297000
H	-7.817514000	-1.856648000	1.203225000
O	8.098933000	2.262754000	-0.199283000
H	8.401178000	2.148783000	0.718305000
O	7.341145000	0.701334000	1.810623000
C	6.987176000	-0.097506000	2.929061000
H	7.672719000	0.179646000	3.740069000
H	5.949137000	0.096794000	3.246756000
H	7.101933000	-1.171372000	2.705717000
C	1.220623000	-1.589189000	-0.577639000
O	1.293406000	-2.712430000	-1.071324000
O	-8.587972000	0.663405000	1.235444000
H	-8.569755000	1.633175000	1.138765000
O	-6.816352000	2.444003000	0.236385000
C	-7.131860000	3.003885000	-1.040675000
H	-7.968799000	2.461261000	-1.511651000
H	-7.424149000	4.048957000	-0.869942000
H	-6.259607000	2.977582000	-1.714266000

Compound 9

C	-5.252945000	-2.708712000	-1.968343000
C	-3.932892000	-2.297886000	-2.135011000
C	-3.258435000	-1.585092000	-1.099196000
C	-3.993956000	-1.340960000	0.081969000
C	-5.312270000	-1.747363000	0.266424000
C	-5.940177000	-2.436271000	-0.773401000
H	-5.756211000	-3.249958000	-2.773905000
H	-5.833708000	-1.530774000	1.198029000
C	-2.165926000	-0.189259000	1.092145000
C	-1.394618000	-0.381684000	-0.021267000
O	-7.221452000	-2.825585000	-0.586125000
H	-7.548234000	-3.288778000	-1.374375000
O	-3.269091000	-2.561196000	-3.280948000
H	-3.858100000	-3.040224000	-3.886555000
O	-3.433787000	-0.675565000	1.128680000
C	-1.868006000	-1.138317000	-1.183820000
O	-1.110850000	-1.372135000	-2.137597000

C	-1.776196000	0.431144000	2.365546000
C	-0.867158000	1.505781000	2.443458000
C	-2.339609000	-0.056854000	3.561971000
C	-0.521111000	2.055820000	3.672151000
H	-0.454388000	1.936816000	1.534862000
C	-1.990919000	0.485970000	4.794008000
H	-3.050553000	-0.883145000	3.526710000
C	-1.071829000	1.544630000	4.857589000
H	0.174646000	2.895194000	3.730129000
H	-2.423446000	0.092224000	5.717471000
O	-0.691824000	2.108729000	6.031925000
H	-1.139100000	1.663643000	6.769340000
O	-0.070248000	-0.011821000	0.014764000
C	0.388759000	0.890328000	-0.955165000
C	1.913505000	0.876742000	-0.916476000
H	0.032508000	0.607070000	-1.957635000
C	0.286939000	3.192879000	-1.499104000
C	2.460844000	1.960735000	-1.830230000
H	2.246396000	1.071613000	0.115362000
C	1.829192000	3.318311000	-1.523496000
H	-0.062465000	2.990715000	-2.529311000
H	2.189837000	1.699767000	-2.871657000
H	2.161212000	3.634079000	-0.516622000
O	-0.107443000	2.169727000	-0.612518000
C	-0.374195000	4.468221000	-1.007432000
O	-1.073914000	4.551316000	-0.024231000
O	-0.061588000	5.502795000	-1.793550000
C	-0.583040000	6.790493000	-1.417860000
H	-0.224175000	7.068851000	-0.417121000
H	-0.209102000	7.495319000	-2.168525000
H	-1.681657000	6.769305000	-1.425274000
O	2.382845000	-0.385214000	-1.370034000
C	2.775692000	-1.278837000	-0.375899000
C	2.707959000	-2.694376000	-0.934092000
H	2.116257000	-1.201060000	0.508187000
C	3.281149000	-3.667590000	0.086488000
H	3.335485000	-2.724513000	-1.845541000
C	4.621824000	-1.780206000	1.042883000
C	4.664557000	-3.236252000	0.553726000
H	2.611869000	-3.662962000	0.969441000
H	3.935936000	-1.735371000	1.911205000
H	5.360246000	-3.298841000	-0.303011000
O	1.358961000	-2.999673000	-1.229322000
H	1.323845000	-3.946841000	-1.433711000
O	3.297941000	-4.954193000	-0.508074000
H	3.651929000	-5.565436000	0.156397000
O	5.052015000	-4.134393000	1.582246000
H	6.015761000	-4.103338000	1.666135000
O	4.104258000	-0.959155000	-0.008498000
C	5.981330000	-1.238703000	1.430641000
H	6.413983000	-1.847338000	2.238305000
H	6.664621000	-1.261811000	0.566858000
H	5.896163000	-0.200954000	1.786005000
O	3.864406000	2.011189000	-1.675736000
H	4.186383000	2.711229000	-2.264358000
O	2.285236000	4.207531000	-2.520368000
H	1.869558000	5.070512000	-2.360999000

Compound 12

C	4.281153000	-0.395422000	-0.000017000
C	3.589864000	0.814455000	-0.000001000
C	2.165016000	0.843406000	-0.000007000
C	1.498424000	-0.407264000	-0.000036000
C	2.174068000	-1.623047000	-0.000050000
C	3.573163000	-1.612451000	-0.000035000
H	5.370506000	-0.370983000	-0.000011000
H	1.623655000	-2.563068000	-0.000069000
C	-0.666042000	0.622121000	-0.000003000
C	-0.086924000	1.862288000	0.000043000
O	4.175631000	-2.820551000	-0.000058000
O	4.251495000	1.991103000	0.000003000
H	5.208127000	1.824394000	-0.000020000
O	0.145867000	-0.479390000	-0.000046000
C	1.364555000	2.050291000	0.000039000
O	1.780601000	3.227231000	0.000077000
C	-2.088372000	0.276857000	-0.000009000
C	-2.484366000	-1.075222000	0.000133000
C	-3.105426000	1.259792000	-0.000158000
C	-3.828052000	-1.444917000	0.000124000
H	-1.727132000	-1.858514000	0.000259000
C	-4.444932000	0.899139000	-0.000172000
H	-2.841238000	2.314042000	-0.000264000
C	-4.823378000	-0.455040000	-0.000037000
H	-4.087786000	-2.502777000	0.000245000
H	-5.226754000	1.661246000	-0.000290000
O	-6.155394000	-0.709919000	-0.000069000
O	-0.783699000	3.024144000	0.000092000
H	-0.075172000	3.709312000	0.000150000

C	-6.594392000	-2.061814000	0.000086000
H	-7.691472000	-2.030132000	0.000067000
H	-6.246295000	-2.597251000	-0.898687000
H	-6.246309000	-2.597035000	0.898994000
C	5.597591000	-2.894127000	0.000022000
H	6.022372000	-2.421001000	0.899906000
H	5.847306000	-3.962510000	0.000002000
H	6.022476000	-2.420941000	-0.899789000

Compound 19

C	3.763374000	-0.867320000	-0.497025000
C	2.557566000	-1.150997000	0.161578000
C	1.647159000	-0.112006000	0.429111000
C	1.975954000	1.200975000	0.033347000
C	3.168997000	1.479632000	-0.630366000
C	4.060716000	0.434462000	-0.905627000
H	4.458750000	-1.686172000	-0.692345000
H	3.394258000	2.502085000	-0.934715000
O	1.076137000	2.218375000	0.231839000
O	5.217727000	0.742680000	-1.559712000
H	5.739833000	-0.064381000	-1.690589000
O	2.338423000	-2.460832000	0.462073000
O	0.438759000	-0.379774000	1.037910000
C	0.918313000	2.650182000	1.585577000
H	1.848283000	3.118266000	1.950055000
H	0.107072000	3.388468000	1.585251000
H	0.644010000	1.815243000	2.246545000
C	1.672181000	-2.833944000	1.668594000
H	0.583441000	-2.873753000	1.532599000
H	2.041670000	-3.837964000	1.922437000
H	1.915961000	-2.143026000	2.489931000
C	-0.724482000	-0.161796000	0.269261000
C	-1.425710000	-1.482323000	-0.023068000
H	-0.450057000	0.321227000	-0.682136000
C	-2.755379000	-1.209208000	-0.712158000
H	-1.624821000	-1.981289000	0.944139000
C	-2.795548000	1.038429000	0.450304000
C	-3.592616000	-0.236374000	0.109889000
H	-2.545919000	-0.754389000	-1.699242000
H	-3.330456000	1.587080000	1.240511000
H	-3.836418000	-0.737275000	1.063435000
O	-1.551738000	0.664550000	1.055439000
O	-0.569945000	-2.263133000	-0.834375000
H	-1.067597000	-3.052861000	-1.096465000
O	-3.417634000	-2.451350000	-0.876833000
H	-4.269361000	-2.262731000	-1.300515000
O	-4.774399000	0.027690000	-0.626234000
H	-5.419888000	0.433999000	-0.029043000
C	-2.610420000	2.000477000	-0.713755000
H	-3.606967000	2.198987000	-1.144431000
H	-1.995374000	1.551017000	-1.512149000
O	-2.009776000	3.184071000	-0.212485000
H	-1.727478000	3.718099000	-0.968237000

Compound 20

C	3.727980000	-1.756702000	-0.112048000
C	2.443320000	-1.811998000	0.439526000
C	1.712897000	-0.652418000	0.666985000
C	2.263224000	0.605558000	0.329069000
C	3.540206000	0.667493000	-0.235946000
C	4.269595000	-0.512872000	-0.451199000
H	4.301046000	-2.669756000	-0.288872000
H	3.993918000	1.618633000	-0.510637000
O	1.484413000	1.684790000	0.580260000
O	5.512528000	-0.379439000	-1.002530000
H	5.912047000	-1.256160000	-1.115233000
O	0.459758000	-0.734335000	1.252016000
C	1.921042000	2.965452000	0.150900000
H	2.059808000	2.992503000	-0.942651000
H	1.128557000	3.667454000	0.434114000
H	2.863209000	3.255670000	0.644214000
C	-0.640777000	-0.391277000	0.441270000
C	-1.558041000	-1.598350000	0.281803000
H	-0.281072000	-0.072941000	-0.551805000
C	-2.825770000	-1.189159000	-0.454701000
H	-1.834301000	-1.942042000	1.296814000
C	-2.477083000	1.154319000	0.430837000
C	-3.482402000	0.002604000	0.232050000
H	-2.545692000	-0.898767000	-1.485320000
H	-2.910410000	1.882499000	1.133534000
H	-3.803943000	-0.331381000	1.234378000
O	-1.318546000	0.651056000	1.106808000
O	-0.850033000	-2.600371000	-0.422565000
H	-1.478653000	-3.315761000	-0.604747000
O	-3.689217000	-2.312943000	-0.483657000
H	-4.493558000	-2.037777000	-0.949963000
O	-4.604926000	0.373310000	-0.549725000
H	-5.173236000	0.944490000	-0.012265000

C	-2.132221000	1.914272000	-0.842936000
H	-3.081654000	2.232740000	-1.306844000
H	-1.616759000	1.265799000	-1.572090000
O	-1.322578000	3.023404000	-0.486743000
H	-0.964950000	3.408099000	-1.299188000
H	1.982629000	-2.768623000	0.691985000

Compound 21

C	-4.273609000	-1.284652000	0.629765000
C	-2.986571000	-1.595475000	0.184661000
C	-2.162737000	-0.605179000	-0.349641000
C	-2.602641000	0.733487000	-0.426794000
C	-3.897489000	1.033101000	0.023574000
C	-4.725215000	0.036761000	0.543335000
H	-4.914457000	-2.066490000	1.042192000
H	-4.235558000	2.069473000	-0.045539000
O	-1.844713000	1.737494000	-0.932759000
O	-0.924022000	-0.986898000	-0.849079000
C	0.215970000	-0.504218000	-0.194974000
C	1.312775000	-1.554612000	-0.249452000
H	-0.025271000	-0.261625000	0.853826000
C	2.610610000	-0.970640000	0.294930000
H	1.464149000	-1.823287000	-1.312277000
C	1.795361000	1.327647000	-0.391857000
C	2.968225000	0.329850000	-0.418610000
H	2.465651000	-0.759265000	1.371220000
H	1.988119000	2.128883000	-1.120372000
H	3.169284000	0.081261000	-1.475387000
O	0.625485000	0.662161000	-0.902254000
O	0.886503000	-2.669358000	0.507702000
H	1.641686000	-3.274767000	0.567685000
O	3.620766000	-1.948806000	0.122214000
H	4.438456000	-1.579116000	0.489565000
O	4.133273000	0.842491000	0.203076000
H	4.523509000	1.509256000	-0.381704000
C	1.549551000	1.983529000	0.958135000
H	2.486018000	2.486726000	1.254558000
H	1.325003000	1.231981000	1.735366000
O	0.478017000	2.897930000	0.805989000
H	0.271676000	3.261207000	1.678784000
H	-5.728942000	0.298223000	0.886449000
H	-2.590563000	-2.611917000	0.234509000
H	-0.906616000	1.451746000	-1.036415000
